# Raising Two More Fundamental Questions Regarding the Classical Views on the Rheology of Polymer Melts

**DOI:** 10.3390/polym16142042

**Published:** 2024-07-17

**Authors:** Jean Pierre Ibar

**Affiliations:** Rheology Department, Polymat Institute, University of the Basque Country (UPV/EHU), 20018 Donostia (St Sebastian), Gipuskoa, Spain; jpibar@alum.mit.edu

**Keywords:** rheology, dual-phase rheology, newtonian viscosity, entanglements, entanglement molecular weight, T_LL_ transition, separation of variables, T and M dependency of viscosity, grain-field statistics, cross-dual-phase

## Abstract

The current paradigm of polymer flow assumes that (i) the effect of the molecular weight of the macromolecules, M, and of the temperature, T, on the expression of the viscosity of polymer melts separate; (ii) the molecular weight for entanglement, M_c_, is independent of T; and (iii) the determination of M_c_ by the break in the log viscosity curve against log M unequivocally differentiates un-entangled melts from entangled melts. We use reliable rheological data on monodispersed polystyrene samples from very low molecular weight (M/M_c_ = 0.015) to relatively high molecular weight (M/M_c_ = 34) to test the separation of M and T in the expression of the viscosity; we reveal that an overall illusion of the validity of the separation of T and M is mathematically comprehensible, especially at high temperature and for M > 2M_c_, but that, strictly speaking, the separation of M and T is not valid, except for certain periodic values of M equal to M_c_, 2M_c_, 4M_c_, 8M_c_, 16M_c_, etc. (period doubling) organized around a “pole reference” value M_R_ = 4M_c_. We also reveal, for M < M_c_, the existence of a lower molecular weight limit, M’_c_ = M_c_/8 for the onset of the macromolecular behavior (macro-coil). The discrete and periodic values of M that validate the separation of the effect of M and T on the viscosity generate the fragmentation of the molecular range into three rheological ranges. Likewise, we show that the effect of temperature is also fragmented into three rheological ranges for T > T_g_: T_g_ < T< (T_g_ + 23°), (T_g_ + 23°) < T < T_LL_ and T > T_LL’_ where T_LL_ is the liquid-liquid temperature. Our conclusion is that the classical formulation of the viscosity of polymer melts is so overly simplified that it is missing important experimental facts, such as period doubling for the separation of T and M, T_LL_, M’_c_, and M_c_, resulting in its inability to understand the true nature of entanglements. We present in the discussion of the paper the alternative approach to the viscoelastic behavior, “the duality and cross-duality” of the Dual-conformers, showing how this model formalism was used to test mathematically and invalidate the separation of T and M in the classical formulation of viscosity.

## 1. Introduction

Polymers are made up of long-chain molecules, which have been studied for more than 70 years, leading to our current understanding of how they flow under stress at high molding temperatures [1]. Viscosity, the stress divided by the strain rate, is the physical variable to follow to describe the effect of the molecular weight of the macromolecules, M, and of the temperature, T, on the ability of the polymer melts to flow in molds, extruders and injection molding machines. Fundamentally, the current paradigm of polymer flow [1] relies on assumptions made at the beginning of the investigations in polymer science that have rarely been questioned, if they ever were:

- Is the separation of the M and T variables in the expression of the viscosity truly validated?
- Is the molecular weight for entanglement, M_c_, truly independent of T, and is the determination of M_c_ by the break in the log viscosity curve against log M, unequivocally assigned to characterize “entanglements”?

The objective of this communication is to provide qualified experimental and mathematical evidence to be able to answer both questions. We analyze reliable rheological data [2,3] on a series of monodispersed polystyrene samples from very low molecular weight (M/M_c_ = 0.015) to relatively high molecular weight (M/M_c_ = 34). The qualification of these data is critically examined. Our tentative conclusions lead to answer no to both questions, yet their generalization will require that the experiments be repeated and extended to include more molecular weight samples. This new **tentative** invalidation of the currently accepted views in the rheology of polymer melts comes after the publication [4] of inconsistencies found in the two most popular dynamic models of the rheology of melts, the Rouse model [5] and the reptation model [6]. 

In the Development section, we show that our viscosity equation explains the appearance of validity of the separation of T and M, especially at high temperature and for M > 2M_c_, but invalidates, strictly speaking, the separation of M and T except for certain periodic values of M equal to M_c_, 2M_c_, 4M_c_, 8M_c_, etc. (period doubling) organized around a “pole reference” value M_R_ = 4M_c_. This paper also suggests, for M < M_c_, the existence of a lower molecular weight limit, M’_c_ = M_c_/8 that we assign to the onset of the macromolecular behavior (the formation of stable macro-coils). The discrete and periodic values of M that validate the separation of the effect of M and T on the viscosity generate the fragmentation of the molecular range into three rheological ranges for M > M’_c_ and/or M > M_c_ as well as the fragmentation of the temperature range above T_g_ into three rheological ranges: T_g_ < T < (T_g_ + 23°), (T_g_ + 23°) < T < T_LL_, and T > T_LL_, where T_LL_ is the liquid-liquid temperature [4,7,8,9,10,11,12]. This fragmentation of the rheological data range challenges the classical claims that molecular motions involving the macromolecules occur identically over the full temperature and that the molecular range is only fragmented into two ranges by M_c_. We introduce in the Discussion section the reasons that led to the formulation of the empirical viscosity equation used to test the separation of M and T and explain how their conception was inspired by the dual-split general concepts and the simulation work specifically done to simulate viscosity [7,8,9].

**Figure 1:**
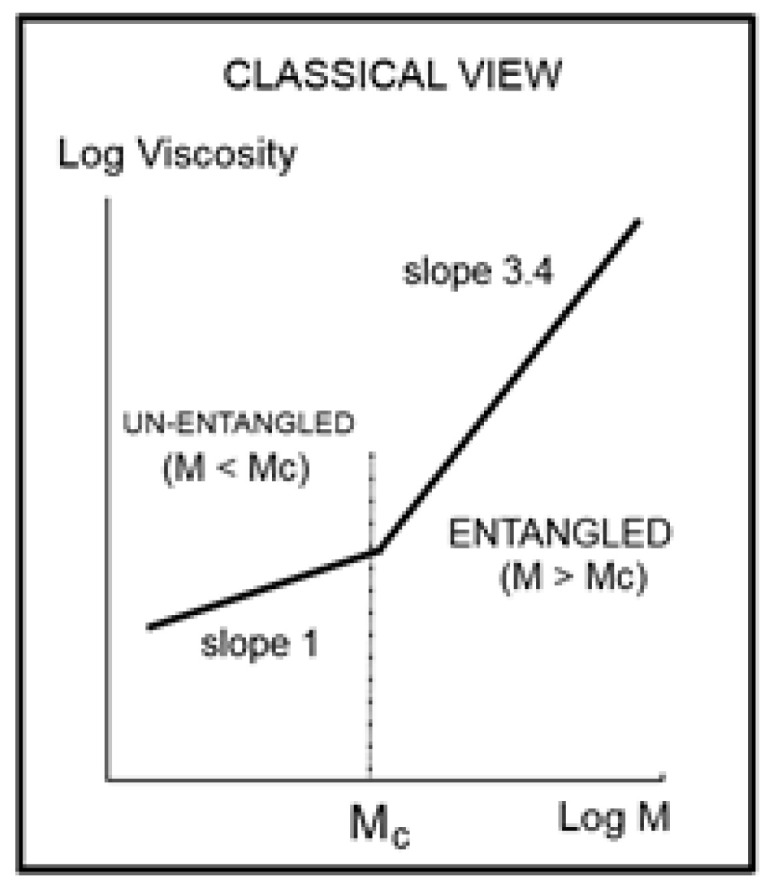
Classical view of the Log of Newtonian viscosity of a polymer melt plotted against the Log of its molecular weight at T constant. Such a plot is shown, for instance, on p.50 (Figure 5.4) of Graessley’s book [1]. **The slope of 1 for M < M_c_ is observed at constant free volume**.

Figure 1 schematically summarizes the classical description of the rheology of polymer melts. Our disruptive new perspective of the effect of M and T to characterize the changes of viscosity presented in this paper reinforces what we already suggested somewhere else [4,10] that the current rheology paradigm has overly simplified the presentation of the experimental facts, perhaps to be able to understand them within the current paradigm framework, and has defensively declared artifacts any experimental deviation from their expectations when there was no possible explanation for those facts by the current paradigm. In our discussion, we briefly present “the duality and cross-duality” of the Dual-conformers, an alternative approach to the viscoelastic behavior of polymer macromolecules. In this new physics of polymer interactions, the Dual-conformers belong to the same macromolecule or different ones and assemble as dissipative open statistical systems of interactions [4,7,8,9]; we conclude that this model explains, at least qualitatively, all the new and provocative results regarding the effect of M and T on the viscosity introduced and analyzed in this paper. 

## 2. Development

The fundamental classical understanding of polymers’ rheology can be summarized in one graph, shown in Figure 1. This plot of the log of the “Newtonian” viscosity (viscosity extrapolated at an infinitely small shear rate) against the log of the molecular weight of the macromolecule, log M, is done at constant temperature or at constant free volume, proportional to T − T_g_, to account for the molecular weight dependence of T_g_ (only affecting the M < M_c_ data since T_g_ is quasi-constant above M_c_). Such a plot is shown, for instance, on p. 50 (Figure 5.4) of Graessley [1]. Figure 1 separates two distinct regions, an “ENTANGLED” region, with a slope of 3.4 for the log μ_o_ vs. log M and an “UN-ENTANGLED” region with a slope of 1. The two regions are separated by M_c_, the molecular weight for entanglements. The large increase in the effect of the molecular weight on the viscosity is remarkably stated by the increase of the slope from 1 to 3.4, which occurs for M > M_c_. The concept of “entanglement” in polymers was derived from figuring out why a sharp break in the viscosity occurred at M_c_ in Figure 1, separating the shorter molecular weights from the longer molecular weights. When the temperature is changed to a different value, Figure 1 remains basically unchanged, except for a vertical shift of the lines, their slopes remaining constant to their respective values across M_c_, and their intersection’s x-coordinate also remaining constant at M_c’_. As a consequence, the Newtonian viscosity μ_o_ is the product of two independent terms, one varying with temperature only, T, and the other one with molecular weight only, M. This is what is designated by “the separation of M and T in the expression of viscosity”. Generally speaking, the experimental evidence backing up the veracity of the features in Figure 1 is overwhelming and has been verified and agreed to by a multitude of qualified peers throughout the literature [1]. The authors of the data that we re-analyze in this paper, Dr. Pierson and Dr. Susuki [2,3] verified and explicitly claimed that their data validated the classical formula, thus the separation of M and T; they compared their data to other researchers’ data, again claiming a complete compatibility. Using a different perspective than classical, this author also examined closely the experimental results in [2,3] and reported in [10] and Chapter 3 of [7], the impact of the measurement uncertainties on the slopes, intercepts, and the determination of M_c_ at their intersection in Figure 1 for the data of Pierson and Susuki. It was reported in [7,10] that what is shown here as Figure 1 was, indeed, “visually observed”, providing the apparent validation of the classical approach. Furthermore, several researchers of the University of Pau and Pays de l’Adour (UPPA) [13,14,15,16] worked on the rheology of monodispersed Polystyrene polymers to study their blends [13,15,16] or validate rheological models based on tube dynamics [15], and compared their results with those done previously by other scientists, explicitly validating their results with those of Pierson and Susuki. In summary, the data of Susuki and Pierson, reanalyzed in the present paper from another angle than the classical rheological approach, are reliable results: they have been verified by us and by several authors who have all claimed the validation of the classical view. The only difference between our own conclusion and these authors’ conclusions was the presence of the word “apparent” before validation in our conclusion. 

**Figure 2:**
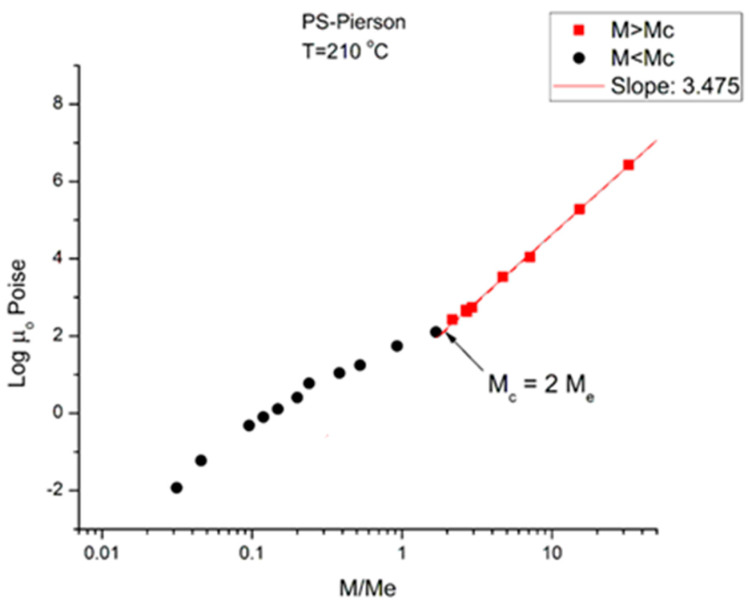
logμ_o_(M) versus M/Meplotted pursuant to Figure 1. Data of Pierson [2] at T = 210 °C.

**Figure 3:**
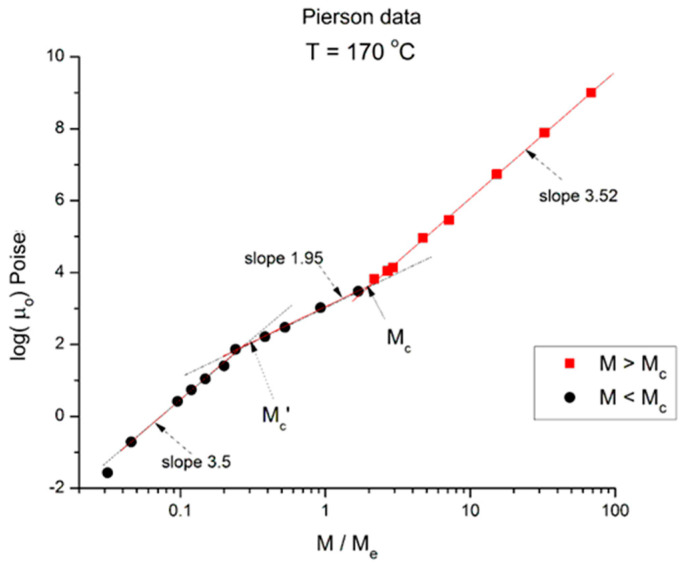
Logμ_o_ (M) at T = 170 °C plotted pursuant to Figure 1. Data of Pierson [2].

**Figure 4:**
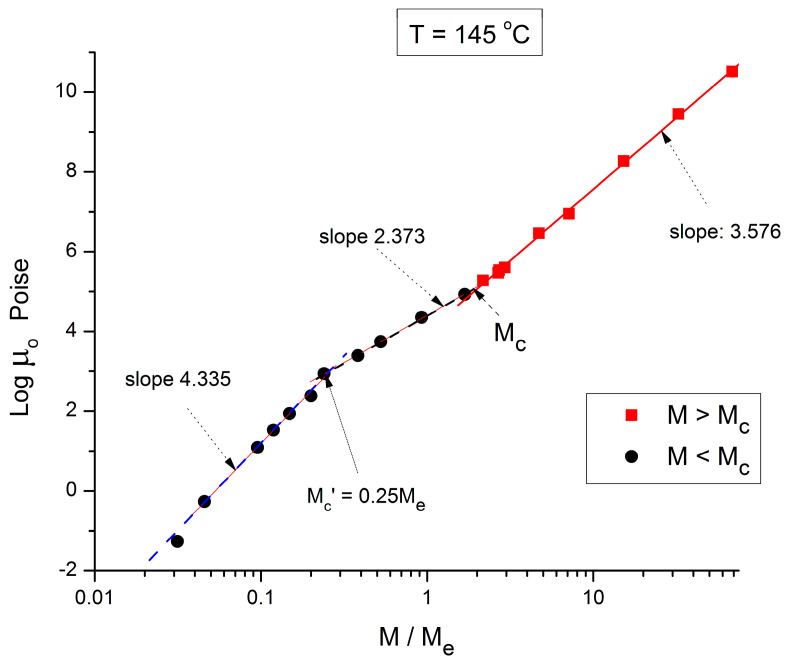
Logμ_o_ (M) data of Pierson [2] at T = 145 °C plotted pursuant to Figure 1.

**Figure 5:**
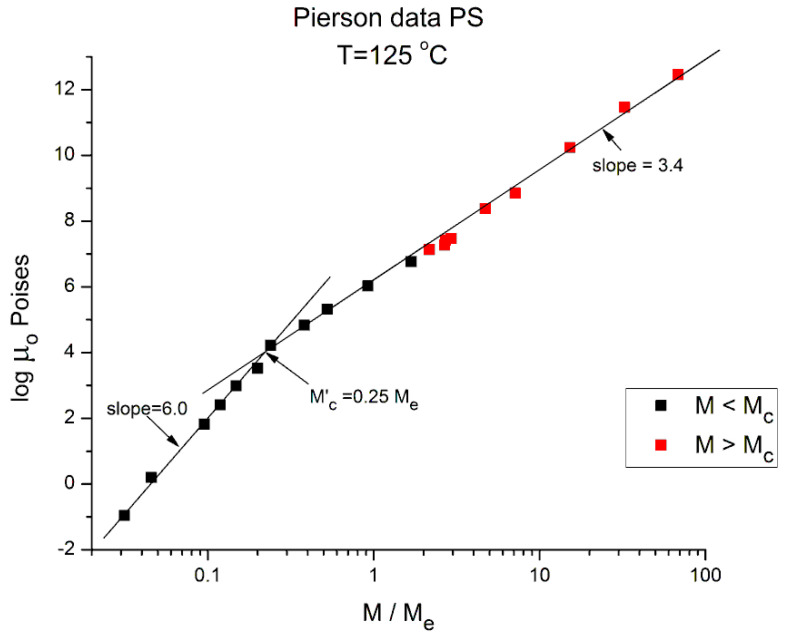
Logμ_o_ (M) data of Pierson [2] at T = 125 °C plotted pursuant to Figure 1.

**Figure 6:**
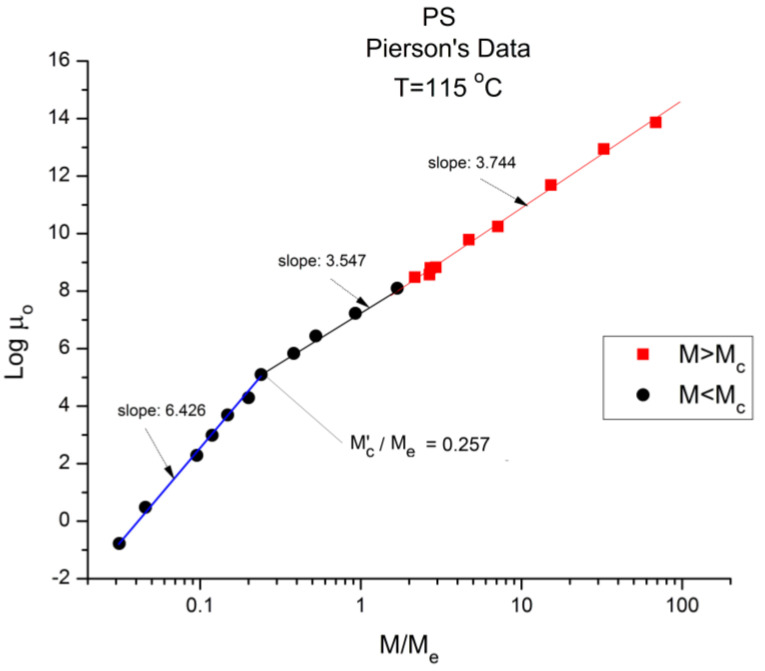
Logμ_o_ (M) data of Pierson [2] at T = 115 °C plotted pursuant to Figure 1. The regressions consider the 2 ranges across M_c_ separate. If we fit one single range starting from M’_c_ the slope is 3.604 (r^2^ = 0.998).

**Figure 7:**
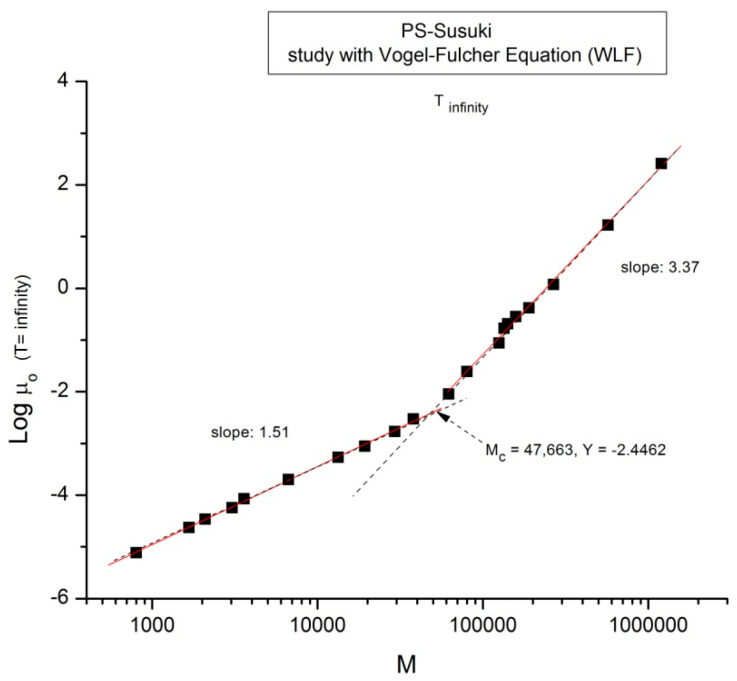
Log μ_o_ (M) data of Pierson [2] at T extrapolated to ∞ and plotted pursuant to Figure 1.

**Figure 8:**
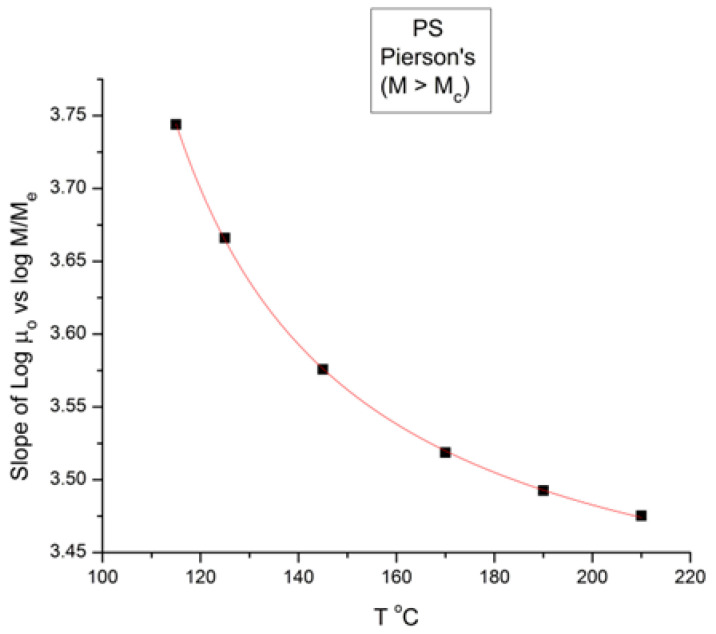
Slope of Log μ_o_ vs. M in the entanglement region (i.e., for M > M_c_) in Figure 1.

**Figure 9:**
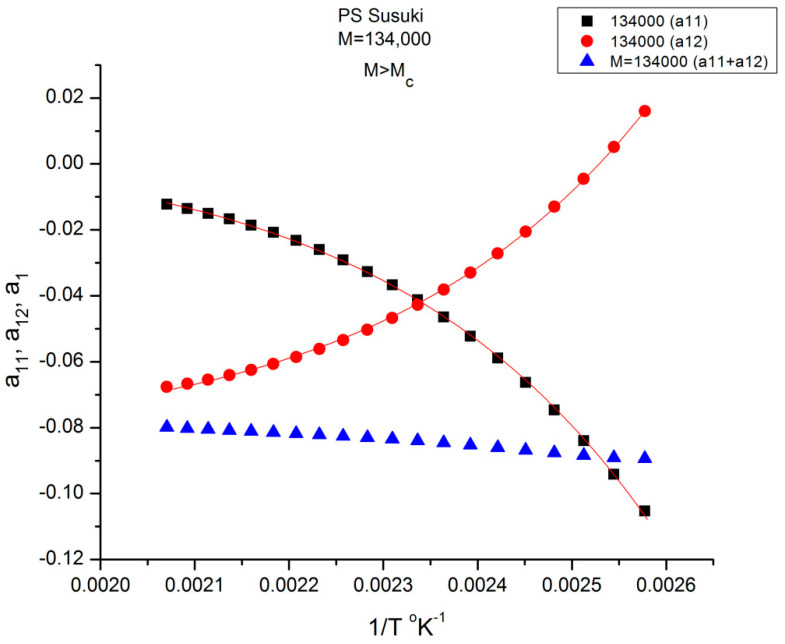
Plot of a_11_(T), the black squares, a_12_(T), the red dots, and a_1_ = (a_11_ + a_12_), the blue triangles, against 1/T for M = 134,000 pursuant to Equations (6) and (7). The red lines passing through the points are the curvefits obtained by regression of Equation (7).

**Figure 10:**
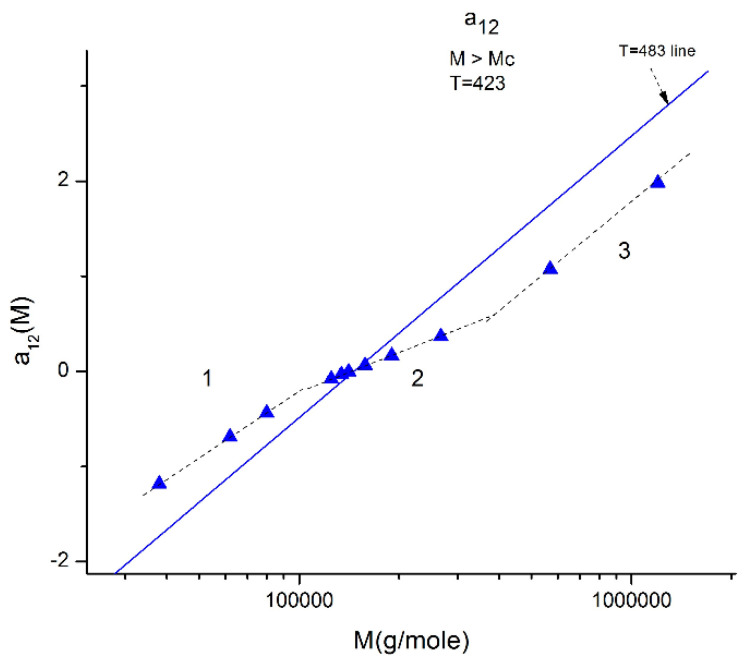
Plot of a_12_(M) at constant T (T = 423 °K is represented by the blue triangles) delimitating 3 ranges: 1, 2, 3. When T varies, the three ranges still exist, but the respective disposition of the three segments is different. The straight line corresponds to T = 483 °K for which the three ranges almost fuse into a single one (range-2 points) is slightly above the straight line yet parallel to it. The T = 423 and T = 483 lines, and the lines for the other T, all cross at a universal “pole R” corresponding to M = 4M_c_ and a_12_ = a_11_ = 0 whatever T.

**Figure 11:**
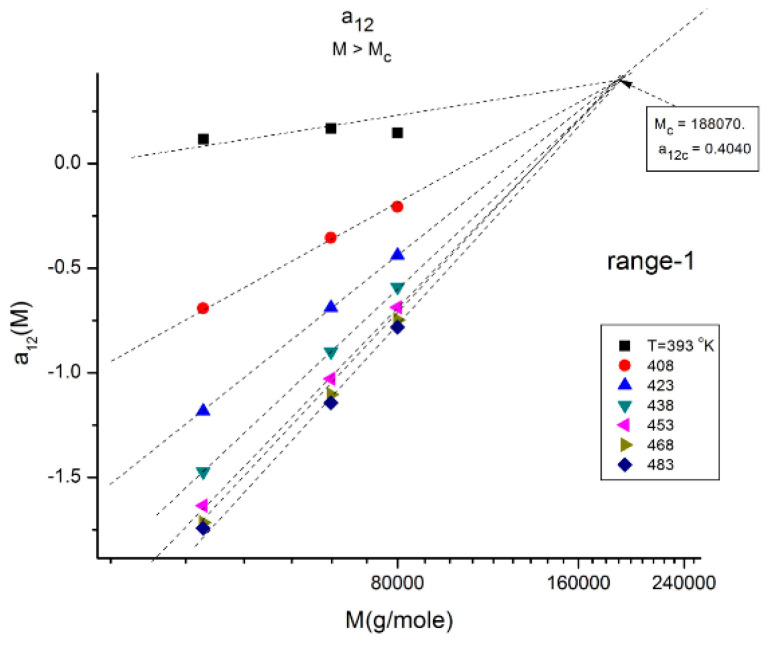
Same figure as Figure 10 restricted to the “range-1” M values and various T. The isothermal segments of a_12_(M) of range-1 all compensate as T increases. The slope reaches an asymptotic limit for T > 453 °K (approximately).

**Figure 12:**
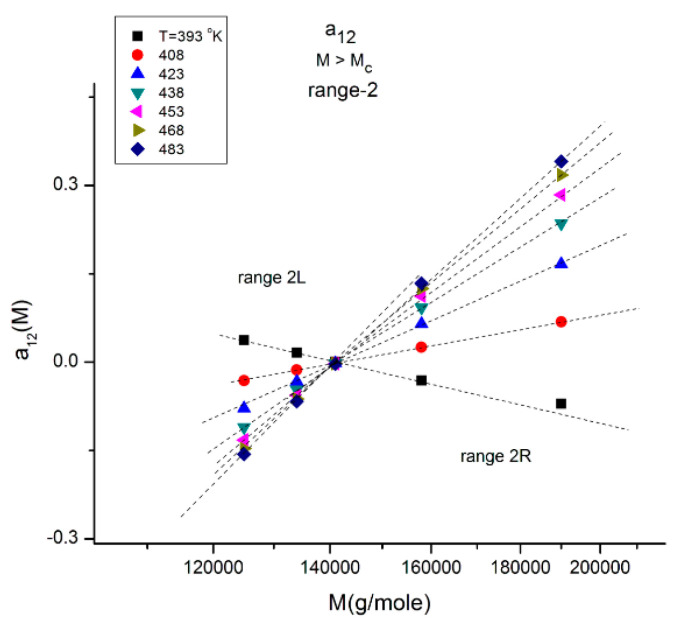
This figure explores range-2 of Figure 10 at various T indicated by the different symbols. There is a double-compensation point at the pole M_R_~4M_c_ delimitating two ranges 2L and 2R The value of a_12_ at the compensation point is zero, which also occurs for a_11_(M) vs. log M (not shown). This figure should be compared to Figure 11 and Figure 13.

**Figure 13:**
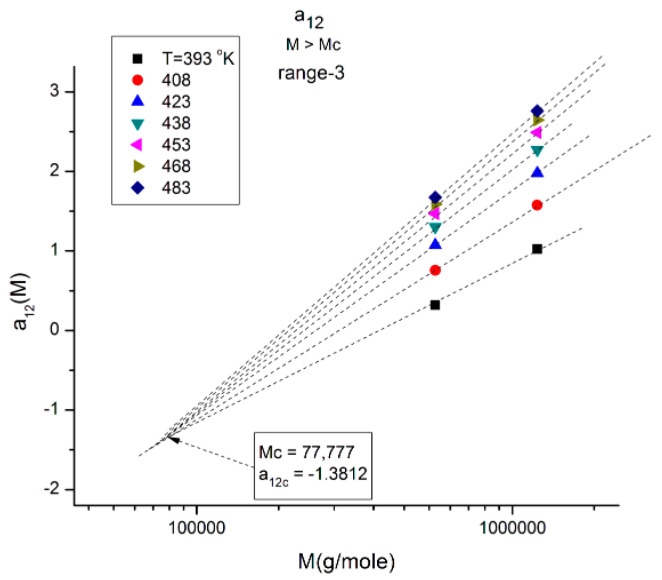
Same as in Figure 10, with a_12_(M,T) now focusing on range 3 and showing a negative compensation point at M~2M_c_. Note the y-coordinate value of the compensation point to be compared with that of the compensation point in range 1 (Figure 11): their sum equals −1.

**Figure 14:**
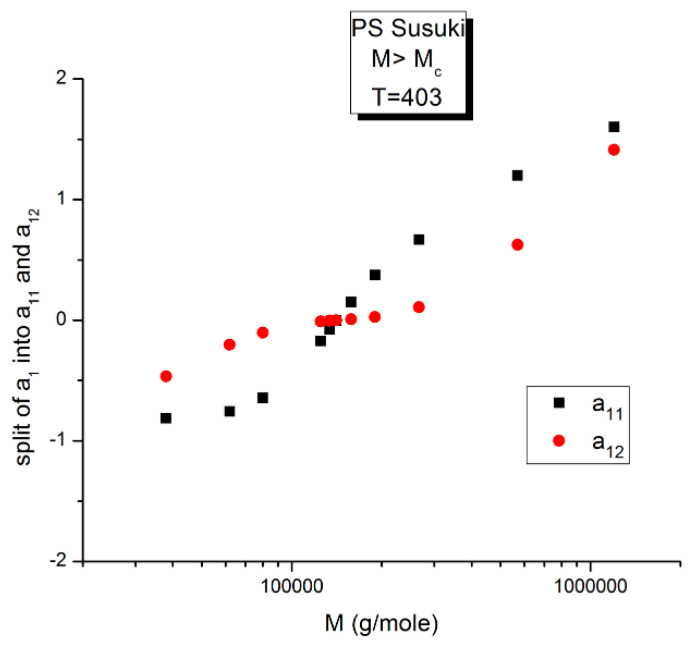
Comparing a_11_(M) and a_12_(M) for T = 403 °K. The a_12_(M) values (red dots) have already been presented in Figure 10, fragmenting the M range into three sub-ranges. The two structural components of a_1_ cross at the pole (M = M_R_), where they become 0.

**Figure 15:**
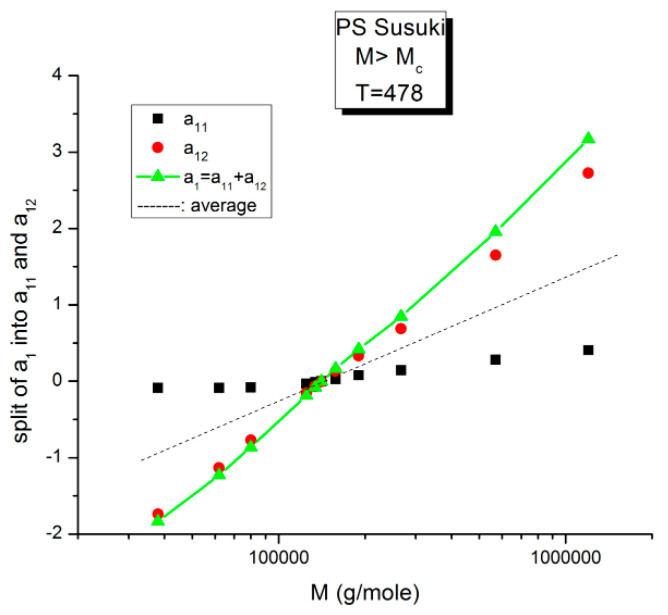
Split of a_1_ into a_11_(M) and a_12_(M) at higher T, 478 K, in this graph. The average value, a_1_/2, represented by the dotted line, appears to be the symmetrical axis of the data, the same across the pole M_R_. The green triangles display the behavior of a_1_(M) vs. log M, which can be fitted by a straight line with slope ~ 3.4, the classical result.

**Figure 16:**
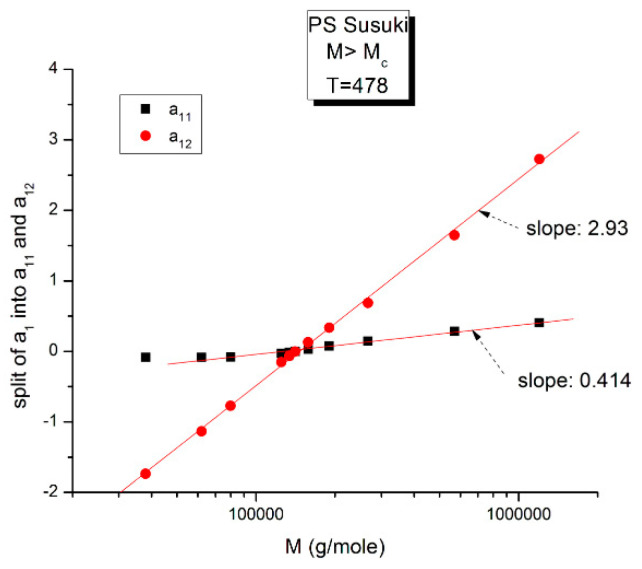
This figure is the same as Figure 15, focusing on different aspects. First, the linearity of the variation of a_11_(M) and a_12_(M) vs. log M for M > M_c_ at higher T and second, the possible significance of the slope of the two “phases” generated by Equations (6) and (7) when plotted against log M.

**Figure 17:**
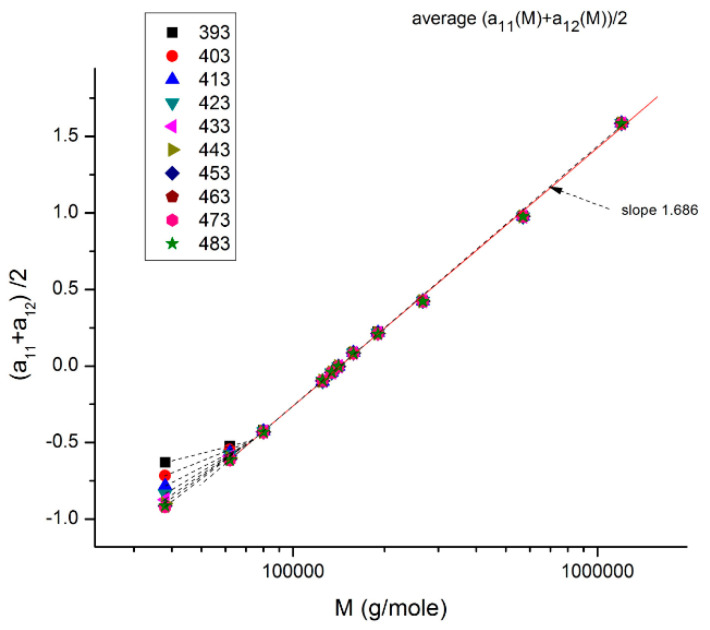
This plot (for M > M_c_) shows the average value between a_11_(T) and a_12_ (T), which is half the value of a_1_ (T) = log μ_o_(T), plotted against log M. For M > 2M_c_ (~77,000), a single straight line goes through the data, which renders μ_o_(T) independent of T: T and M separate. Below 2M_c_, we observe a compensation of lines as T varies instead of the unique straight line: T and M no longer separate.

**Figure 18:**
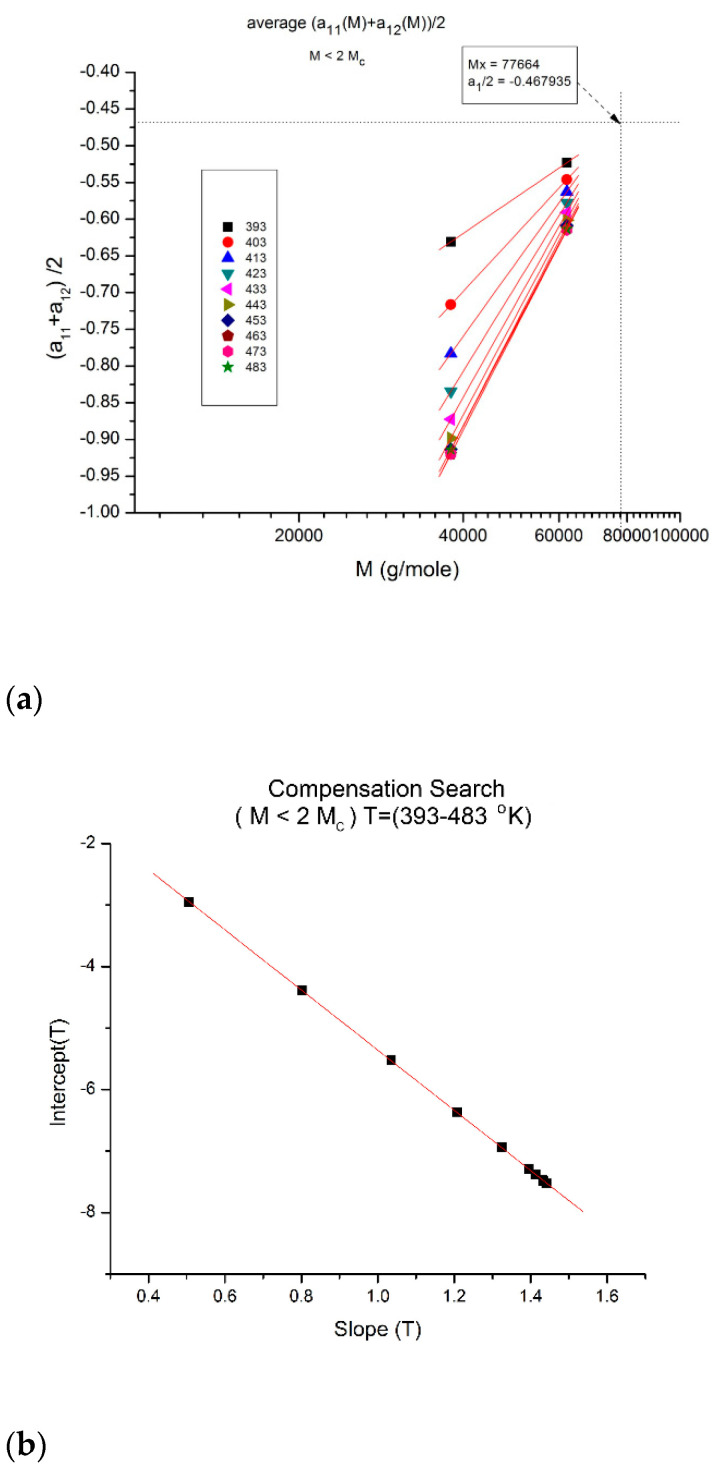
(**a**) Compensation of the isothermal segments for the average of a_11_(M) and a_12_(M) in range-1 (M < 2M_c_). The compensation Search is displayed in (**b**). (**b**) Compensation search to determine the compensation point coordinates in Figure 18a; the coordinates are calculated from the slope and intercept of the compensation line in this plot: intercept:−0.4679; slope: −4.89022. The value of M at the compensation point is, therefore, 10^4.89022^ = 77,664, and the y-coordinate is −0.4679.

**Figure 19:**
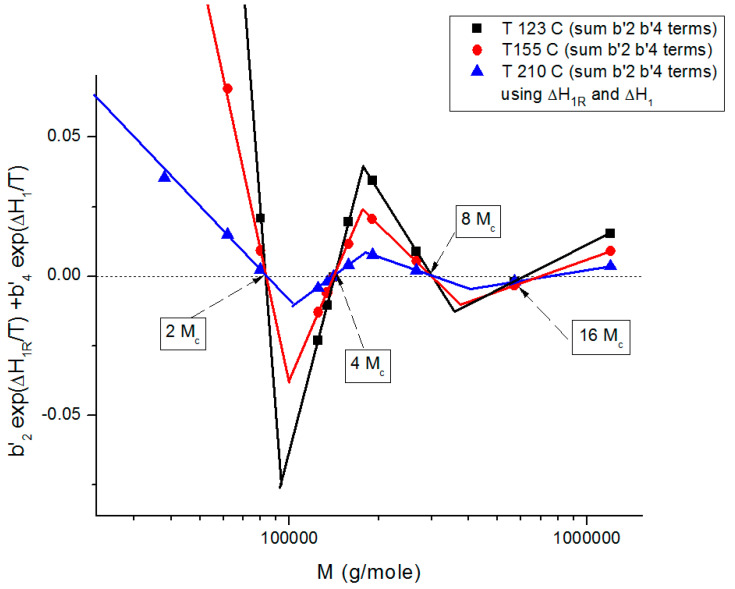
Proposed structuration by the coupling of T and M towards variable separation, which occurs at M = 2M_c_, 4M_c_, 8M_c_, 16M_c_ for configuration (0,1) in Equation (7). This figure shows the alignment of the data points on straight lines, their compensations occurring on the zero horizontal line and the systematic departure from the criteria for the separation of M and T whose amplitude gets smaller as M increases (M > 2M_c_), explaining the appearance of the M and T separation more real at higher M values. See [8] for details.

**Figure 20:**
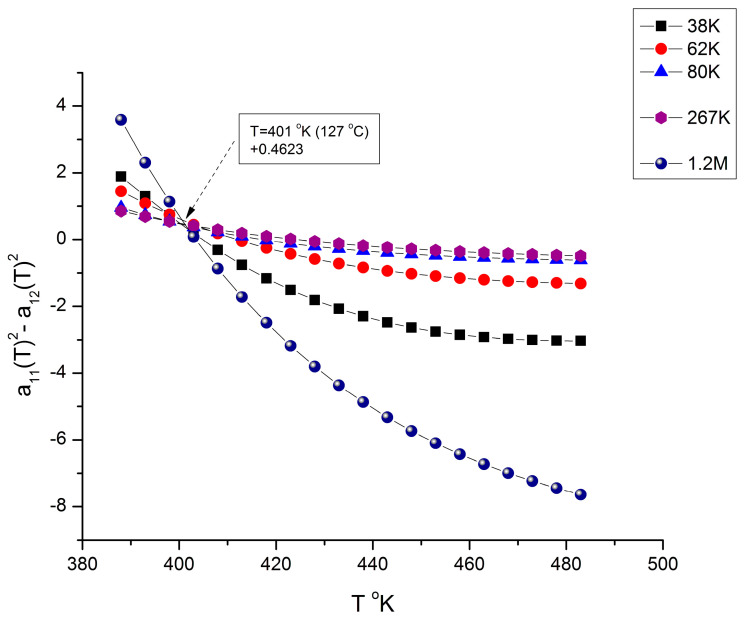
The data a_11_(M,T) and a_12_(M,T) are now plotted against T at constant M (the reverse of Figure 11, Figure 12 and Figure 13).

**Figure 21:**
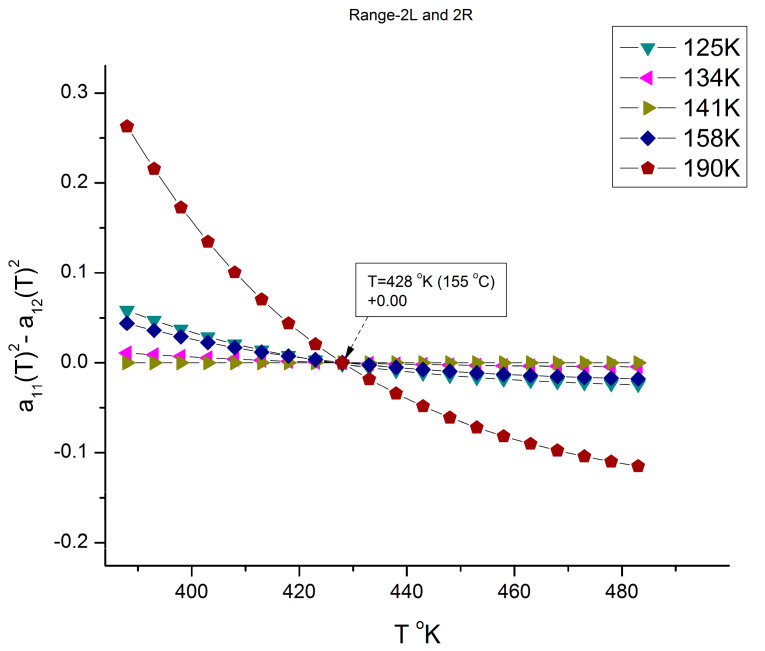
This figure is complementary to Figure 20 and shows the same plot but for different values of M, all belonging to range-2. All the curves indicated compensate at T ~ T_LL_. Also notice that the y-coordinate of the compensation pole is 0, a situation also occurring for M_R_ in Figure 11, making these two “transitions” related [8]. Note: in the insert, 125K refers to M = 125,000, etc.

This paper is divided into three sections: in the first section (A), illustrated by Figure 2, Figure 3, Figure 4, Figure 5, Figure 6, Figure 7 and Figure 8, we analyze Newtonian viscosity data plotted like in Figure 1 at various temperatures, showing that the classical picture of the effect of T and M on the viscosity and on the definition of M_c_ depicted by Figure 1 may be incomplete and too simplistic to be practically useful and leads to the wrong conclusions regarding the nature of entanglements. In the second section (B), illustrated in Figures 9–21, we introduce a new empirical formulation of the viscosity (inspired by the Cross-Duality concept) to address the testing of the separation of M and T. This mathematical formula quantitatively describes the viscosity data “perfectly” (r^2^ = 1.0) when M and T vary and can be plotted like in Figure 1 to determine the slopes projected by the classical formulation of viscosity (Figure 1). This mathematical fitting exercise reveals the invalidation of the separation of M and T except at values of M corresponding to period doubling multiples of M_c_. We also conclude in section (B) on the futility of finding a theoretical explanation for the value of the slopes, 1 and 3.4, respectively, for M < M_c_ and M > M_c_ in Figure 1. Finally, in the last section (Discussion), we point to the main differences between the admitted polymer dynamic models’ approach to mechanical flow and our disruptive model of polymer interactions that is manifested by a new interpretation of the viscoelastic effects and entanglements.

A. The Effect of Molecular weight on the Newtonian Viscosity at various constant T.

Figure 2, Figure 3, Figure 4, Figure 5, Figure 6 and Figure 7 plot Log (μ_o_) vs. Log (M/M_e_) data obtained at various temperatures for a series of monodispersed Polystyrene (PS) samples. μ_o_ is the Newtonian viscosity (in Poises), and M_e_ is the molecular weight between entanglements, known to correspond to ~M_c_/2 where M_c_ demarks the break between the un-entangled and entangled data in Figure 1. The data were first generated and analyzed by Pierson in his PhD thesis at the University of Strasbourg [2]. The same monodispersed PS samples were part of an extended study and retested by Susuki [3], also at the University of Strasbourg, revealing an excellent repeatability of the results of Pierson and a concurring analysis using the classical formula of rheology. As already mentioned, Pierson and Susuki’s viscosity results on monodispersed PS were tested and validated over the years by several scientists at the university of Pau and Pays de l’Adour (UPPA), for instance, by Majeste [16], Montfort [13], Marin [14], and Cassagnau [15]. The Susuki’s data will be used in section B of the paper because it involves more samples with M > M_c_. Theses authors adhered to the established practice of the separation of the effect of T and M during their analysis. This provides an excellent example of rheological data that can be analyzed with an “apparent great satisfaction” according to the classical views, yet with great concerns when our approach analyzes the same data from a different angle. For instance, Pierson and Susuki use the Vogel-Fulcher equation and the WLF equations (Equations (1) and (2)) to express the temperature dependence of the friction factor of the Newtonian viscosity at constant M.

Logμ_o_ = A + B/(T − T2) (1)

where A, B, and T_2_ are fitting parameters that vary with M [1,17]. This equation can be rewritten with respect to a temperature reference, for instance, T_g_ at which the viscosity is μ_og_:

log (μ_o_/(μ_og_) = −C_1g_(T − T_g_)/(C_2g_ + (T − T_g_) (2)

with

C_1g_ = B/(T_g_– T_2_) and C_2g_ = (T_g_ − T_2_) (3)

where the fitting constants, C_1g_ and C_2g_ are often called the WLF constants [1,17]. The viscosity is now expressed as a function of the free volume, proportional to (T − T_g_). The validity of the Vogel-Fulcher and the WLF equations over the temperature range is restricted by the classical models to hold between T_g_ to (T_g_ + 100°); beyond that, Equation (1) is often replaced by a thermally activated expression providing an Arrhenius description of the temperature dependence of the Newtonian viscosity Chapter 11 of [17]. The fragmentation of the rheological data into two ranges has been critically reviewed by this author in several publications [10,11,12], Chapter 3 of [6]; it is also an important issue raised in the present communication.

Figure 2 corresponds to the data of Pierson [2] at T = 210 °C plotted pursuant to Figure 1. The unit for viscosity is Poise (i.e., 0.1 Pa-s). The x axis is log (M/M_e_) where M_e_ ~ M_c_/2. The value of M_e_ is equal to *ρ*RT/G_N_ pursuant to the theory of rubber elasticity where *ρ* is the melt density; R is the gas constant; T is the absolute temperature; and G_N_ is the plateau elastic modulus of an entangled melt [1,4]. We use red square symbols for M > M_c_ and dark dots for M < M_c_. Above M_c_, the data conform with what is classically expected: the log-log plot is a straight line with slope = 3.475 and intercept 1.164.

Below M_c_, the log-log plot of the data should be a straight line with slope 1 according to classical predictions; instead, the dark dots align according to a curve more or less smoothly. This curvature, apparently contradicting the prediction of the Rouse model of the Newtonian viscosity proportionality to M for un-entangled polymers (M < M_c_), has been reported and explained by several authors [1,10,16,17], chapter 3 of [6]. In particular, it was pointed out, as an explanation, that working at constant free volume instead of constant temperature would re-establish the proportionality between μ_o_ and M [1,10,17]. It is clear in Figure 2 that the general understanding of the effect of increasing the molecular weight conforms to what is predicted by the molecular dynamic models: 1. the longer the chains, the more difficult it is to shear them; 2. “entanglement” results in a significant increase in the difficulty to process polymer melts because of the increase of the viscosity by a ratio at least equal to M^2.4^ (i.e., M^3.4^/M), which is the ratio of the viscosity of an entangled melt to the viscosity of the same melt without entanglements present (a so-called disentangled melt) at the same temperature and for the same molecular weight M. 

We now examine the same data but plotted at other temperatures T = 170 °C in Figure 3, T = 145 °C, 125 °C, 115 °C in Figure 4, Figure 5 and Figure 6, respectively. Finally, Figure 7 provides the value of the Newtonian viscosity versus M when T becomes infinity in the Vogel-Fulcher equation: T = ∞ → Logμ_o_ = A. 

Essentially, Figure 3 can be compared to Figure 2 by saying that it is the same figure with a new look at considering the M < M_c_ data, in particular in the way the un-entangled range is broken down into two log-log lines instead of assuming it was a smooth curve in Figure 2. In fact, in the other Figure 4, Figure 5 and Figure 6, we follow this attempt to break down the low molecular weight region and compare the changes occurring to the slopes of the log-log straight lines thus defined.

In Figure 3 we have three straight line “sections” with slope, from left to right: 3.5, 1.95, and 3.52. The molecular weight delimitating the sections are M’_c_, between range 1 and 2, and M_c_ between range 2 and 3, respectively. We see that (M’_c_/M_e_) ~ 0.25 and (M_c_/M_e_) ~ 2. The slope for the entangled data (the red squares) is not very different at T = 170 °C and at T = 210 °C, and the difference may look insignificant, proving that the separation of the effect of M and T would hold in this region. However, a fine study of this effect shows that the slope not only does not remain constant but systematically varies with T in a small yet continuous way (see Figure 8). The slope of 3.5 found for the first seven lower molecular weights is as big as that found in the entangled region. Why would the viscosity of low molecular weight “un-entangled” PS samples increase with M like M^3.5^, an exponent thought to be characteristic, precisely, to the presence of entanglements?

Figure 4 applies to T = 145 °C, showing the three regions and their respective slopes, from left to right: 4.335, 2.373, and 3.576, respectively. The breaks occur at M’_c_ = 0.25 M_e_ and M_c_ = 2 M_e_. Figure 5 and Figure 6, for T = 125 °C and 115 °C, respectively, are different because they only reveal two log-log straight lines, not 3. The originality is the absence of the break at M = M_c_! The slope in region 1, for M < 0.25 M_e_, continues to sharply increase as T decreases, 6.0 for 125 °C, 6.38 for 115 °C. The slope in region 3, for M > M_c_, continues to increase slightly as T decreases: 3.61 for T = 125 °C, and 3.63 for T = 115 °C. As already mentioned, this slope is not independent of T, as it should be to validate the separation of M and T in the formulation of the Newtonian viscosity. It is remarkable that Figure 5 and Figure 6 show no entanglement break from M = 0.25 M_e_ to 100 M_e_, raising legitimate concerns regarding the concept of entanglement and its origin. 

Additionally, the meaning of the slopes of Logμ_o_ vs. Log M themselves raise questions, including in the entanglement section of M. The traditional approach considers the change of the value of the exponent, typically from 1 to 3.4, to be important, even essential, to characterize the molecular weight dependence of viscosity. What about if this exponent turns out to be the wrong variable to tell what happens to the statistical properties of a set of macromolecules when their molecular weight increases? 

Take Figure 7 for instance: it is a plot of Log μ_o_ vs. Log M for T = ∞ in the Vogel-Fulcher equation. This figure could be used as a perfect illustration of the teaching of the classical views regarding entanglements: two regions of M are visible, well separated, and well described by the classical tools, with a slope of 3.37, almost perfectly 3.4, above M_c_, and a slope of 1.5 below M_c_. Although the slope below M_c_ is not 1, with possible reasons given before, the break between the high and low molecular weights is sharp and well defined. The problem, though, is that the M_c_ value at this sharp break, M_c_ = 47,663, is significantly greater (+36%) than the M_c_~ 35,000 classically assigned to the molecular weight for entanglements for PS, p.55 [1]. Figure 2 correctly shows M_c_ at 35,000 for the same data. Would this be a sign to indicate that M_c_ varies with T, which it should not do according to the classical paradigm? Figure 5 and Figure 6 show the break in the viscosity curve occurring at 0.25 M_e_, not 2M_e_ as projected by the classical explanation of entanglements. One sees that using the break in the viscosity curve to define M_c_, like it is illustrated in Figure 1, is not coherent with the claim that M_c_ does not vary with temperature: Figure 5, Figure 6, and Figure 7 provide clear evidence that the value of M_c_ varies with T when it is defined by the break of the log viscosity-logM curve (Figure 1). An alternative explanation is based on the instability of the entanglement state [18], resulting in the possibility of manipulating the value of M_c_, refer to the chapter 8 of [7]. 

Finally, there is the question of the meaning of M’_c_~0.25 M_e_ = M_c_/8 = 4375 g/mole. This molecular weight stands out in all the figures, separating the very low M and the region below M_c_. Both Pierson [2] and Susuki [3] admit the existence of a deviation from the standard analysis in this region without raising the possibility of a molecular transition. To the best of our knowledge, the recognition of such a deviation as a true “molecular transition” below M_c_ has never been reported in the literature, except recently while analyzing T_LL_ (M) in Ref. [12]. The molecular weight of a monomeric repeat unit for PS is 104.15 g/mole, and M’_c_ corresponds to 42 such units. As T decreases from Figure 2, Figure 3, Figure 4, Figure 5, Figure 6 and Figure 7 from T infinity to T = 115 °C, respectively, the slope of log μ_o_ vs. M in the region M < M’_c_ monotonically increases from 1.5 to 6, a four-fold increase of the exponent of the effect of M on viscosity. It is clear that the admitted validation of the separation of the effect of M and T on viscosity badly fails in this region.

Figure 8 is a plot of the slope of log μ_o_ vs. M in the entanglement region (i.e., for M > M_c_) in Figure 1. The classical value of the slope is 3.4, as said earlier, yet Figure 8 shows that, although the value of the slope is not far from 3.4, it is not constant when T increases and, even more problematically, it decreases monotonously, not randomly, from 3.635 at T = 115 °C to 3.475 at 210 °C, a clear indication that the exponent does not strictly remain constant when T varies. As we reported earlier, such a small change in the slope is not easily visually perceptible, giving the illusion of a constant slope for M > M_c_ when T varies. A regression analysis performed in the M > M_c_ range at various T unequivocally produces a small but systematic non-randomness of the deviation from 3.4 (or from whatever average value is chosen). Figure 8, already presented in a previous paper [10], was our first exposure to the possible failure of the separation of T and M in the expression of the viscosity, an indication that motivated the research presented in this communication. The second section of this paper, section B, will quantitatively explain the conditions rendering the effect of M and T on the viscosity separable. 

We conclude from Figure 2, Figure 3, Figure 4, Figure 5, Figure 6, Figure 7 and Figure 8 that the basic foundation of the classical understanding of the effect of molecular weight and temperature on the Newtonian viscosity of polymer melts (Figure 1) is possibly contradicted by the above observations. To pursue this idea that the classical views might have oversimplified the effect of M and T in the formulation of the melt viscosity of polymers, we present in section B a mathematical demonstration that allows us to determine whether the effect of M and T on the viscosity do separate or not.

B TESTING MATHEMATICALLY THE SEPARATION OF M AND T IN THE EXPRESSION OF VISCOSITY.
  B1 Summary of the classical approach.

For polymers of low molecular weight (M < M_c_) in Figure 1, the Newtonian viscosity takes the form:

μ_o_(M,T) = K(T) M   (M < M_c_) (4)

where M is the molecular weight of the chains, μ_o_ is the Newtonian viscosity at temperature T, and K is a constant that varies with (T − T_g_) to make the free volume constant in Equation (3). Note that the slope of 1 in Figure 1 for M < M_c_ requires in Equation (4) that K(T) be actually K(T − T_g_) to work at free volume constant, where T_g_ is a function of M below M_c_ (i.e., T_g_(M)). This means that the classical viscosity equation below M_c_ admits that M and T do not separate. This should be pointed out.

For longer chains (M > M_c_), the classical and quasi-universal value of 3.4 reflects the power law dependence of the entanglements on the viscosity:

μ_o_ (M,T) = K’(T) M^3.4^    (M > M_c_) (5)

The critical molecular weight, M_c’_ is obtained from intersecting the straight lines Log μ_o_ vs. Log M drawn in the two regions M < M_c_ and M > M_c_. 

Formula (5) simply states that molecular weight and temperature effects separate in the expression of the viscosity of polymer melts. Since T_g_ is constant for M > M_c_, K’(T − T_g_) does not make this term function of M, unlike for M < M_c_. The temperature dependence of K(T) or K’(T) in Equations (4) and (5) is often written, as we already said, with the WLF expression in Equation (3). The WLF equation explains the curvature observed in Arrhenius plots of log(μ_o_) vs. 1/T and is claimed to work well between T_g_ and T_g_ + 100, with the reservations made previously regarding the veracity of that statement. The constants C_1g_ and C_2g_ in Equation (3) are often admitted to have the universal value of 17.44 and 51.6, respectively, a statement discussed and refuted in a previous communication [10]; in fact, it was shown that C_1g_ and C_2g_ are not constant when M is varied and that their molecular dependence revealed the fragmentation of the rheological range, an observation that will also be forthcoming from the Dual-Phase approach presented next.

B2 The Dual-Phase Inspired Approach.

Let us consider Equations (6) and (7), inspired by the classical formulation of rheology, yet modified pursuant to the two-phase nature of the Dual-conformers. This formulation of the effect of M and T on the Newtonian viscosity is empirical, as much as Formulas (4) and (5) are also empirical. The main difference between the classical empirical formula, (4) and (5) and the Dual-Phase inspired empirical formula, (6) and (7), is in the interpretation of the physical parameters entering the equations. This will be explained in the Discussion. In the following, we refer to “phase 1” and “phase 2” or their respective Newtonian viscosity, μ_o1_ and μ_o2_ pursuant to our assumption of a possible “split” of Logμ_o_ into a structure: Logμ_o1_ and Logμ_o2_, corresponding to (μ_o_ = μ_o1_*μ_o2_). The split is expressed in the first line of Equation (6):

(6) $$\begin{array}{l} \mathrm{Log}\mu_{o}(T,M)=a_{1}(M,T)+\mathrm{Log}\mu_{o}R(T)=\mathrm{Log}\mu_{o1}(T,M)+\mathrm{Log}\mu_{o2}(T,M)\\ a_{1}(M,T)=a_{11}(M,T)+a_{12}(M,T)\\ \mathrm{Log}\mu_{o1}(T,M)=a_{11}(M,T)+\mathrm{Log}\mu_{o1}R(T)\\ \mathrm{Log}\mu_{o2}(T,M)=a_{12}(M,T)+\mathrm{Log}\mu_{o2}R(T)\\ \mathrm{Log}\mu_{o}R(T)=\mathrm{Log}\mu_{o}(T,M=M_{R}) \end{array}$$

where the sub-index “11” refers to phase 1 and “12” to phase 2. When a_1_(M,T) = a_1_(M), Log μ_o_(T,M) expresses the separation of M and T:

(7) $$\begin{array}{l} a_{12}=b_{1}+b_{2}\exp(\Delta H_{2R}/T)+b_{4}\exp(\Delta H_{2}/T)\\ a_{11}={b’}_{1}+{b’}_{2}\exp(\Delta H_{1R}/T)+{b’}_{4}\exp(\Delta H_{1}/T) \end{array}$$

where ΔH_2R_ and ΔH_1R_ are constant (the value of ΔH_2_ and ΔH_1_ at M = M_R_, respectively), ΔH_1_ and ΔH_2_ are a function of M. The values of b_1_, b_2_, and b_4_ and b’_1_, b’_2_, and b’_4_ can be set to 0 or split into two terms (e.g., b_1_ = b_1_(a_11_) + b_1_(a_12_), b’_1_ = b’_1_(a_11_) + b’_1_(a_12_) etc.), which may both vary with M or T, M and T, or remain constant, for instance to 0. The testing of the different assumptions generates a matrix of testing possibilities, called “configurations”, that are exhaustively studied but not presented in this first presentation on the subject. For each set of assumptions, ΔH_2R_, b_1_ b_2_ b_4_ and ΔH_2_ or ΔH_1R_, b’_1_ b’_2_ b’_4_ and ΔH_1_ are found by non-linear regression of the data.

When b_2_ and b_4_ simultaneously equal 0 in Equation (7) or when (b_2_exp(ΔH_2R_/T) +b_4_exp(ΔH_2_/T)) = 0 and then a_1_(M,T) = a_1_(M) = b_1_ and there is true separation of the variables M and T. Same thing for the b’_2_ and b’_4_ possibility. For the sake of this mathematical demonstration, there is no need to attribute any physical meaning to the split of the viscosity into two separate components. One can consider the split, at this stage, as a possible way to improve the fit of the experimental values in a way similar to the improvement of the fit of the melt modulus E(t) by using two relaxation terms or even a spectrum of relaxation terms instead of a single relaxation term (Maxwell or Voigt models [10]). As we already said, the physical interpretation of the “Dual-Phases”, phase 1 and phase 2 will be explicit in the Discussion. 

Equations (6) and (7) were used to fit the PS monodispersed Newtonian viscosity data of Susuki [3] for M > M_c_. We generated a set of “synthetic data” from the data in [3] in order to increase the number of T and M pairs, and the following analysis in the paper relates to the synthetic data. The procedure used to obtain the synthetic data was as follows: the original Susuki data were first curve-fitted with Equations (3) or (4), depending on the value of M. The viscosity values were re-tabulated every 5° from 110 to 210 °C for each M, providing a matrix of 420 values. The matrix was then transposed to obtain the same data but with a different line-up to permit plots of Log(μ_o_) vs. Log(M) at the same temperature T. The synthesized data were re-analyzed by non-linear regression for each T, using Equations (6) and (7). The regressions results were statistically “perfect” (r^2^ = 1 with random residuals) for all combinations of T and M, validating its use for our purpose of testing the separation of M and T. Note the absence of bias in the starting equation, in the sense that the possibility to find the classical assumption of a full separation for all M and T is included in our validation test matrix. The results of the tests are described in Figure 9, Figure 10, Figure 11, Figure 12, Figure 13, Figure 14, Figure 15, Figure 16, Figure 17 and Figure 18b corresponding to test configuration (0, 0), the simplest formulation for M > M_c_. Another example of configuration, (0, 1), will be given in the Discussion (Figure 19), among the four configurations possible in the matrix of tests. The complete analysis will be published separately [8]. 

Figure 9 is a plot of the values of a_11_(T) and a_12_(T) found by regression for M = 134,000 according to Equations (6) and (7). The black squares represent a_11_, and the red dots represent a_12_. The blue triangles refer to their sum a_1_(T) = a_11_(T)+ a_12_(T) in Equation (6). The validation test of the separation of M and T consists in determining whether the sum a_1_ = a_11_+ a_12_ of Equation (6) remains constant when 1/T varies (and the blue triangles remain horizontally aligned) for this particular value of M. Figure 9 shows that the horizontality of a_1_ is never realized for M = 134,000 since a_1_(M) slightly varies with T. The slope is closer to 0 in the higher T region (the lower 1/T values). The separation of M and T is invalidated for this molecular weight (134,000 g/mole). We repeat the same test in Figure 9 for all the other M values available. For certain discrete values of M, e.g., 2M_c_, 4M_c_, 8M_c_, etc. the line of blue triangles remains strictly horizontal when T varies: a_1_(M,T) = a_1_(M) for those discrete values of M. For the other values of M, a “structure of a_1_(M) appears visible, fragmenting a_11_(M,T) and a_12_(M,T) into ranges of sequential M, for which compensations occur. It appears that the compensation points always coincide with the values of M for which the blue lines are horizontal. Depending on the configuration status of our assumptions in Equation (7), some period doubling compensation may be missing (e.g., 16M_c_ for configuration (1,0) [8]).

We illustrate these observations in Figure 10, Figure 11, Figure 12, Figure 13 and Figure 14 by plotting a_12_(M) vs. log M for three fragmented ranges of M,T values. Figure 10 shows the separation of the molecular weight range in three sub-ranges, all above M_c_, for a_12_(M) at T = 423 K (150 °C).

In Figure 11, the values of a_12_(M,T). for M_c_ < M < 2M_c_) (i.e., in “range-1”), at various T form a collection of straight lines compensating at M = 188,000 ~5M_c_ when M is plotted on a log scale. The y-coordinate of the compensation point is positive. The lines compensate as T increases from 120 °C to 210 °C. The slope of a_12_ (T) vs. log M reaches an asymptotic value in the higher T region, which lines up this range-1 of data with the data located in the other ranges, range 2 and range-3, at higher M (Figure 12 and Figure 13).

Figure 12 explores “range-2” of the values of the molecular weight, showing a compensation point at M = M_R_ = 144,000 ~ 4M_c_. The value of a_12_ at the compensation point is zero, which is also the case for a_11_ (not shown), indicating that a_1_ = a_11_ + a_12_ = 0 is a special solution for the separation of M and T corresponding to a_11_ = a_12_ = 0. In Figure 12 we see that in range-2 (M < M_R_), the compensation lines converge to the compensation point, whereas they emerge from the compensation point in range 2R (M > M_R_).

In Figure 13, the a_12_(T,M) values of M are located in range-3, and when T varies, the isotherms compensate at M~2M_c_ with a negative value of the y-coordinate of the compensation point. Interestingly, if one compares the three ranges in Figure 11, Figure 12 and Figure 13, the higher M range (range-3 in Figure 13) corresponds to a “negative” compensation; that is, the isotherms emerge from a lower M value, while the compensation is “positive” in range-1 (Figure 11), i.e., the isotherms converge to a higher M value. The situation in range-2 is intermediate with the combination of a positive and a negative compensation using the same compensation point at M = M_R_. The description of a_12_(M,T) can thus be described as a succession of positive and negative compensations, a very interesting network structure threaded by the interdependence of the effect of M and T on viscosity. Also noticeable are the complementarities of the y-coordinate values of the compensations for Figure 11 and Figure 13, which add up to −1. 

The variation of a_11_(M,T) is similar to a_12_(M,T) for the effect of M at T constant, yet almost symmetrically “opposite” to a_12_(M,T) for the effect of T at M constant. In Figure 14, Figure 15 and Figure 16, a_11_ and a_12_ are plotted against log M at T constant, showing two temperatures only: a low T = 403 °K (130 °C) and a high T = 478 °K (205 °C).

We see in Figure 14, for this low T value (T = 403 °K), the appearance of symmetry for a_11_(M) and a_12_(M) around their mean value, but in reality, for such low temperatures up to T = 448 °K, the axis of symmetry is different for the values of M superior or inferior to M_R_ = 4M_c_(at M_R_, both a_11_and a_12_ are equal to 0, as we saw in Figure 12). Data points are missing at both extremities of the plot to confirm the possibility for a_11_(M) and a_12_(M) to cross on the symmetry lines at M ~ M_c_ and M~55.5 M_c_, confirming the presence of periodic anchors for discrete M values.

Figure 15 displays the equivalent of Figure 14 at a higher temperature: T = 478 K (205 °C). Like at lower T, a_11_(M) and a_12_(M) cross at M_R_ = 4M_c_, but the disposition of the red dots (a_12_) and of the black squares (a_11_) on the graph looks different than at lower temperatures. This implies that the influence of T on the interdependence of M and T is not the same in the three sub-ranges of M, range-1, 2, or 3. Likewise, the influence of M on the interdependency of T and M is not the same in the three temperature sub-ranges 1, 2, or 3, delimited by T_g_, T_g_ + 23 °C and T_LL_ (see the discussion). The green triangles represent a_1_(M) = a_11_(M) + a_12_(M) vs. log M (i.e., a plot of log μ_o_ vs. log M pursuant to Equation (6)). These points can be fitted by a straight line across M_R_ (r^2^ = 0.9983) with slope = 3.372, which is the classical exponent value characteristic of PS melts. This is expected since we used data generated by the classical formula (Equations (3–(5)). One sees that a two-phase formulation of the viscosity, Equations (6) and (7), that assumes viscosity to be the product of two terms for which the effect of M and T are not separable, provides the same value of the slope for log μ_o_ vs. log M for M > M_c_ than in the classical view, that assumes that the effect of M and T on the viscosity separates (Figure 1). This apparent paradox proves, at least, that the veracity of the features in Figure 1 does not uniquely imply the separation of M and T in the viscosity. On the contrary, Figure 10, Figure 11, Figure 12, Figure 13 and Figure 14 demonstrate the particularities of the dependence of the effect of T and M on the viscosity in ways that cannot be deducted if M and T were separable. In particular, among the special features of the dependence between M and T is the finding of a special molecular weight M_R_ = 4 M_c_. Figure 15 shows the average value of a_11_(M) and a_12_(M), a_1_/2, represented by the dotted line, which appears to be the symmetrical axis across this M_R_ value. This “pole” molecular weight, a clear marker of the dual-phase viscosity formulation in range M > M_c_, is not M_c_ itself but 4M_c_, a value of M that does not resonate to be significant in the classical paradigm. Likewise for the T scale in the T > T_g_ range, as we further explain in the Discussion section, the T_LL_ transition [11,12] has no physical reason to exist in the classical theories of melt viscoelasticity and is vehemently described as an artifact by the adepts of the classical views [12], yet erroneously so, in our opinion, since this temperature determines the success or the failure of a new processing benefit for the polymer resins industry: “sustained orientation” [4,7]. 

Figure 16 may possibly be the simplest explanation of the classically acclaimed formulation of viscosity by Equation (5) for M > M_c_. A Dual-Phase interpretation of the classically acclaimed 3.4 exponent is that 3.4 = (3 + 0.4) and that one should separate the understanding of the exponent 3 from the exponent 0.4, instead of trying to modify the explanation of the exponent 3 (say by de Gennes [5]) by incorporation of additional mechanisms such as contour length fluctuation, constraint release and chain stretching (Chapter 7 of [7]) to reach the value of 3.4 for the exponent. A Dual-Phase explanation is the separation of the phases in the viscosity equation instead of the separation of the effect of M and T.

Figure 16 is the same as Figure 15 plotted differently, showing different aspects of this graph: the variation of a_11_(M), the black squares with slope 0.414, and of a_12_(M), the red dots with slope ~3 at high T. The difference between the lower temperature range of the melt (e.g., in Figure 10) and the higher temperature range (Figure 16) is now clearer: the lines in Figure 16 represent the log μ_o1_(M), and the log μ_o2_(M) vs. log M viscosity plots of the two-phases 1 and 2 at T = 478 °K, and these lines are straight all across and not fragmented segments as they appear at lower T (403 °K in Figure 15). This, again, clearly reveals not only the separate and distinct role of M and T on the viscosity but also their compensating interactive effects.

Speaking in terms of viscosity, Figure 16 represents the molecular weight dependence of the log of viscosity of “phase 1 and phase 2” pursuant to the “Dual-Phase equations”, Equations (6) and (7): it provides the dual-structure of log μ_o_ into two terms, log μ_o1_, represented by the black squares for a_11_(M) with slope 0.414 (phase 1) and log μ_o2_, the red dots for a_12_(M), with slope 2.93 (phase 2). Note that if we change the temperature from T = 478 °K to T = 483 °K, the respective slopes of the lines in Figure 16 become 0.328 and 3.0 for the respective phases, giving a total exponent of 3.328 for the variation of a_1_(M) = log μ_o_(M) in Equation (7). It is, indeed, remarkable to find for the slope of phase 2, the “core-phase”, the value of 3, which is the value found by de Gennes in the original reptation calculation of the molecular weight dependence of the Newtonian viscosity [6] and to attribute to phase 1 “the entanglement phase” the “corrective” additional 0.4 exponent value classically assigned to sophisticated perfections of the original de Gennes’ tube calculations (see Figure 16 caption). 

Figure 17 illustrates how the separation of M and T for M > 2M_c_ is simply the result of an illusion created by the use of log axes that compresses the resolution of a significant residual periodically fluctuating around 0. This figure plots half the value of log μ_o_ = a_1_ against log M for all the T values available (T = 393–483 °K). We observe that a_1_(M)/2 = (a_11_ + a_12_)/2 varies with T and M in the lower molecular weight range (M_c_ < M <2M_c_), but that in other ranges of M (M > 2M_c_) there is a single straight line with all the points superposing; that is, it *apparently* looks temperature independent, varying only with M; the effect of M and T appears to separate in the expression of the total viscosity for M > 2M_c_. In this region of M and T, the slope of a_1_ vs. log M is 2 * 1.686 = 3.372, the expected value from the classical model of viscosity. Below M = 2 M_c_, however, we observe a spectrum of compensating lines as T varies, the visible proof using these axes that T and M at least do not separate in this range. The details for M < 2M_c_is blown up in Figure 18a, and the compensation search is shown in Figure 18b. We can clearly see that the slope of each isotherm increases with T up to T ~ 428–448 °K (155–175 °C) and that for T beyond that temperature, the slope asymptotically converges to a value close to (but not equal to) the 3.372 slope of the T independent region (Chapter 4 of [8]).

In range-2 and 3 for M, the a_11_(T) and a_12_(T) are more symmetrically disposed with respect to their average value when M varies, especially at high temperatures (e.g., Figure 15), and therefore their half sum gives the illusion to lie more closely on the average value line, giving the appearance of being independent of T. However, if we zoom on the points of the M > 2M_c_ region in Figure 17 or if we analyze the residuals of their linear regression, the illusion of the separation of T and M reveals itself unequivocally (see Figure 12 and Figure 13). 

Another interesting observation consists of comparing the compensation coordinates for the a_1_(M,T) data in range-1 (Figure 18a) and for the a_12_(M,T) data in range-3 (Figure 13): they have the same value of M at the compensation but their y-coordinates are shifted by approximately 1, a clear indication that the fragmentation of the T range and the M range for T > T_g_ and M > M_c_, respectively, are dependent and coupled in the expression of the viscosity of polymer melts, an important conclusion totally missed by the current paradigm of rheology.

In summary, the regression of the Newtonian viscosity data as a function of T and M by Equations (6) and (7) show that b_2_ and b_4_ become simultaneously zero for periodic values of M, which are multiples of M_c_ (33,000 to 36,000 g/mole), pursuant to period doubling: 2M_c_, 4M_c_, 8M_c_, 16M_c_. For all other values of M, the values of b_2_ and b_4_ are quite small but not zero, yet small enough to make the sum (b_2_exp(ΔH_2R_/T) + b_4_exp(ΔH_2_/T)) give the appearance that the separation of the variables is justified, although it is not. Furthermore, we observe that the sum (b_2_exp(ΔH_2R_/T) + b_4_exp(ΔH_2_/T)), although small, structures with M in a way that makes the periodic molecular weights for the zeros the compensation points for the other M values when M is expressed as log M (see the Discussion at Figure 19 for a visual summary). As we mentioned at the onset of section B, our current presentation of the periodic dependence of T and M in the expression of the viscosity has been simplified in this paper. A more complete and thorough mathematical proof will be provided separately [8]. The key result of this section B is the understanding that all current models of melt viscoelasticity of polymers based on the separation of M and T are approximations that overly simplify the effect of M and T, miss out some fundamental aspects of the interactive coupling between T and M, and are, as proven mathematically, incorrectly describing the interactions in polymers, theoretically speaking. Of course, it should also be added that the separation of T and M becomes a better approximation at higher melt temperature and for M > 2M_c_ (Figure 17), which are the usual ranges of use of polymer resins, a choice that may explain why the previous models [4,8] remained falsely convinced of the true separation of T and M, despite of the contrary in reality.

## 3. Discussion

To summarize the results presented in sections A and B, we conclude that Figure 1, representing the concepts of the current paradigm of rheology, is incomplete and misleads the comprehension of the flow of melts because it is too simplistic; it does not correctly describe the effect of temperature, T, or the effect of the molecular weight, M, and misses out the consequences of their coupling on the fragmentation of rheological ranges affecting the Newtonian viscosity; in many ways its shortcomings become inconsistencies leading to false interpretations. For instance, the classical interpretation of the viscosity break at M_c_ as the proof for entanglements (Figure 1) appears to be directly challenged by the work presented in this communication (Figure 2, Figure 3, Figure 4, Figure 5, Figure 6 and Figure 7). The failure of the classical interpretation of entanglements has been reported and documented by us before [4,7,18], showing its negative impact on the polymer processing industry [4,7]. This paper makes use of mathematical tools developed from ideas inspired by the dual and cross-dual aspects of the rheology of polymer melts [7,8,9] to reveal fundamental inconsistencies in the current assumptions leading to the classical understanding of the rheology of polymer melts [10]. 

In section A, the graphs in Figure 1, Figure 2, Figure 3, Figure 4, Figure 5, Figure 6 and Figure 7 encompass the M < M_c_ and M > M_c_ regions to evaluate the classical view assumption that the entanglement molecular weight is defined at the crossing of the distinct behaviors below and above M_c_. Figure 1 schematizes the break at M_c_ that is supposed to be independent of T. In the other graphs, in Figure 2, Figure 3, Figure 4, Figure 5 and Figure 6, the change of color for the squares, from red at high M to black at lower M, starts at the value of M_c_ projected for PS: 33,000 to 36,000 g/mole [1]. The highest T is in Figure 7 (T = infinity), followed by, in descending order, Figure 2, Figure 3, Figure 4, Figure 5 and Figure 6. Figure 7 is the only graph resembling Figure 1: 2 straight lines and a sharp break at M_c_, accepting the reservations we made for the 1.51 slope for M < M_c_ (instead of 1) and the higher value of M_c_ at the break than the quoted values in the literature (48,000 instead of 36,000). Only Figure 2 (at T = 210 °C) could validate with a certain certitude the classical view for M_c_ and above M_c_, yet needed the reserves we made for the description of the M < M_c_ distinct curvature from the expected straight line one in Figure 1. M_c_ is at its correct literature value in Figure 2. As T decreases, from Figure 3 to 6, the overall aspect of the graph starts to systematically diverge from the classical expected view of Figure 1, displaying three straight lines (not two) in Figure 3 and Figure 4, along with two molecular breaks, M_c_ and M’_c_, not just M_c_; and then, only two straight lines with a break at M’_c_ and no break at M_c_ for the two lowest T (Figure 5 and Figure 6). For instance, in Figure 5 (T = 125 °C), there is no break at the change of color from red to black at M_c_, and the straight line passing through the “entangled melt” continues unbroken down to M’_c_ = M_c_/8. Is the entanglement molecular weight now defined by M’_c_ = M_c_/8? Do we need to define another criterion than the break of the molecular weight in a graph like Figure 1 to characterize M_c_? This is the first fundamental challenge raised in this paper to the current paradigm of rheology. Along with it, what is the physical significance of the M_c_/8 molecular weight?

The second fundamental question raised concerns the separation of the effect of M and T in the formulation of the viscosity. This question already surfaces in Figure 8, at the end of section A, by showing that the molecular weight exponent for the Newtonian viscosity, the famous 3.4 slope in Figure 1 for M > M_c_, explained with great details by the reptation school [6], seemingly varies *systematically* with temperature, which would contradict the separation of T and M if it were confirmed. Section B presents a simplified version of a mathematical proof that we have developed [8] to evaluate the classical assumption of the separation of T and M in the formulation of viscosity. The overall conclusion is that the separation of T and M in the expression of viscosity is invalid except for a series of specific molecular weight values that are multiples of M_c_: 2M_c_, 4M_c_, 8M_c_, 16M_c_, etc. We found that the value of M_R_ = 4M_c_ holds a special role among all the solutions, the role of a “pole reference” for the melt (a_11_ = a_12_ = a_1_ = 0), symbolized by the presence of the subscript “R” in some of the terms in Equation (7). In other words, we discovered in section A the existence of a certain special molecular weight M’_c_ = M_c_/8, and in section B the existence of another special molecular weight, M_R_ = 4 M_c_, possibly indicating that the period doubling observed above M_c_ could actually start at M’_c_? Note that playing along with this period doubling picture, the value of M_e_ of the rubber elasticity theory falls right between M’_c_ and M_c_ with M_e_ = M_c_/2 = 4 M’_c_. Figure 19 summarizes the period doubling fragmentation of the M range at various values of T pursuant to “configuration (0,1)”. Figure 9, Figure 10, Figure 11, Figure 12, Figure 13, Figure 14, Figure 15, Figure 16, Figure 17 and Figure 18a,b above referred to configuration (0,0), the simplest to describe. The difference between the configurations bears on the complexity and the status of certain constants to be found by regression, which results in certain solutions being skipped for period doubling: for instance, 16M_c_ is not a solution for configuration (1,0). These complications are not essential for this introductory presentation.

Figure 19 shows the sum of the b’_2_ and the b’_4_ terms in Equation (7), configuration (0,1)), plotted against log M. This graph summarizes the various facets of the sophistication of the coupling between the T and M variables introduced in the previous figures (Figure 9, Figure 10, Figure 11, Figure 12, Figure 13, Figure 14, Figure 15, Figure 16, Figure 17 and Figure 18b): it shows the true values of the zeros (b’_2_ = b’_4_ = 0) corresponding to the compensation of the non-zero regions, it shows the fragmentation of M in three regions and the period doubling of M_c_. The low value of the sum of the b’_2_ and b’_4_ terms in Figure 19 explains the visual illusion of the separation of M and T perceived in Figure 17. Also, the sum of the b’_2_ and b’_4_ terms becomes smaller for M > 2M_c_ and as T increases, making the separation of T and M appear more real.

We mentioned in several instances of this presentation and in previous publications [7,10] the need to fragment the melt temperature range in 3 rheological ranges for T > T_g_, in a way similar to the fragmentation in three rheological ranges of the M molecular range in Figure 10, Figure 11, Figure 12, Figure 13 and Figure 19: M’_c_ < M < M_c_, M_c_ < M < M_R_, and M > M_R_. The fragmentation of the temperature delimitates T_g_ < T < T_g_ + 23 °C, T_g_ + 23 < T < T_LL_, and T > T_LL_ where T_LL_ is the liquid-liquid temperature associated with the Dual-Phase model’s dissipative transition temperature [4,7,8,9,10,11,12]. T_LL_ varies with M [11] but takes values between 155 to 175 °C for PS. T_g_ is also variable with M below M_c_ reaching an asymptote in the M > M_c_ range so that the value of T_g_ + 23 °C for M > M_c_ stabilizes around 125 °C. [10]. Figure 20 and Figure 21 give a possible explanation for the temperature fragmentation of the melt above T_g_ using the mathematical framework of Equations (6) and (7).

Figure 20 and Figure 21 show the same plot (which could have been combined in one graph, yet they are split for clarity to show their different poles for selected different values of M). Figure 20 only has the selected group of M values shown in the insert that belong to either range-1 or range-3, but none of range-2 of the molecular weight fragmentation. The other values of M giving the alternative pole are shown in Figure 21. All the curves in Figure 20 compensate at T ~ T_g_ + 23 °C., and all the curves in Figure 21 compensate at T ~ T_LL_. Note: in the insert, 38K refers to M = 38,000, etc.

Both Figure 20 and Figure 21 are plots of (a_11_^2^ − a_12_^2^) vs. T for various values of M remaining constant when T varies. In a certain sense, these figures are similar to Figure 11, Figure 12 and Figure 13, which displayed the fragmentation of the M range in three sub-ranges. Figure 20 and Figure 21, however, use a slightly different y-coordinate, and the role of M and T is reversed: T varies, and M is constant in Figure 20 and Figure 21, whereas M varies and T is constant in Figure 11, Figure 12 and Figure 13. Yet the presence of poles (compensations) is visible when T is scanned from T_g_ to 210 °C. In Figure 20, the curves compensate at T = 401 °K (127 °C), but only for a discrete set of M values listed, not for all the values of M (the other values of M are in Figure 21); additionally, the compensation y-coordinate in Figure 20 is not 0, but 0.4623. Figure 21 applies to all the M values not listed in Figure 20 (i.e., for M = 125,000 to 190,000), and it is shown that the curves compensate at a different temperature, T = 428 °K (155 °C), and on the y-coordinate axis (a_11_ = a_12_ = 0). The values found for the two compensations, 127 °C and 155 °C in Figure 20 and Figure 21, respectively, appear to match the value given for T_g_ + 23 °C and T_LL_ from other estimation sources [9,10,11,12]. In other words, the fragmentation of the temperature and of the molecular weight ranges into rheological sub-ranges is due to the interactive coupling between the effect of M and T in the definition of the viscosity (shown as an example in Equations (6) and (7)).

Figure 20 and Figure 21 reveal an added complexity, which is absent in Figure 11, Figure 12 and Figure 13: whether the curves, for any given value of M, belong to either the pole group in Figure 20 or Figure 21. This complexity in the inter-coupling between M and T, and of its incidence on the fragmentation, has been eluded to earlier when we noted that the existence of pole 16M_c_ was part of the period-doubling solutions for configuration (0, 1) in Figure 19, but not for configuration (1,0). This shows that the rheology of the coupling μ_o_(M,T) is, indeed, more sophisticated than what has been introduced in this communication and needs more elaboration [8].

Let us now provide a short presentation of the differences between our challenging model of polymer physics to explain the rheology of polymer melts and the classical molecular dynamic models of rheology currently admitted as the current paradigm. The reader should refer to the Preamble of [7,18] as well as [4] for a brief presentation of the theoretical background of our new model of interactive dissipative coupling applied to polymers [7,18] and of its practical application to “disentangle” polymer melts during processing to temporarily decrease their viscosity during processing [4]. These three references are fully available from the ResearchGate archive platform (“Preamble: Introduction to the Dual-Phase Model of Polymer Interactions and to the Cross-Dual-Phase Model of Entanglements”).

The present understanding of the physics of macromolecules is based on an analysis of the properties of a single chain. The presence of the other chains is perceived as a mean field influence on the properties of that chain. The reptation model of de Gennes [6] considers that “entanglements” arise at M_c_ when the homogeneous field, creating the environment of the chains, is modified due to the higher molecular weight of the macromolecules to include obstacles restricting the motion of the single chain. In other words, topological constraints occurring for higher molecular weights chain lengths create the obstacles that explain the rise of the viscosity at M_c_ compared to the viscosity in the absence of such obstacles, which has been modelized by Rouse [5]. For the classical view of rheology, the relaxation time of the molecular motions occurring within the chain, in the presence or not of the topological constraints, generates the stresses and strains when the fluid is submitted to a strain rate tensor to put it in motion. The challenge is to correlate the relaxation times obtained from the analysis of the rheological parameters, for example, from the Newtonian viscosity or the cross-over relaxation time to the relaxation times calculated from the conformation and configuration description of the polymer chain.

In conclusion, to briefly summarize the foundation of the classical dynamic models of rheology [5,6], their statistical system is the macromolecule, and the chain dynamic is described by the chain relaxation time (Rouse or reptation time).

**Figure 22:**
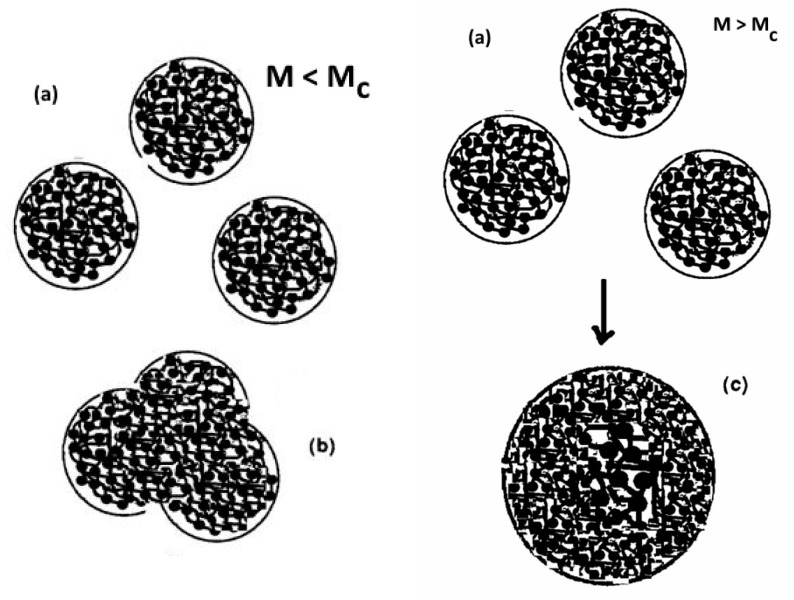
Left: cartoon representation of the single Dual-Phase system where the dual-phases are represented by the b-grains (black balls) surrounded by white space representing the F-conformers. Assuming that single macromolecules can take the shape of “macro-coils”, represented by the larger spheres, which they can do for M > M’_c_, (3 macro-coils are represented in (a), their interpenetration remains a single Dual-Phase as long as M remains below M_c_, which is shown in (b). Right: when M > M_c_, the interpenetration of the macro-coils as a single Dual-Phase is instable and results in the split into two interlaced (“entangled”) single Dual-Phases (c), which are constantly redefining their interface to remain stable, generating the presence of an elastic dissipative shear wave in the molten state [7,18].

For the Dual-conformers approach, the statistical system studied to define the stresses and the strains is not a single chain, and the molecular motions are not apprehended by their relaxation times. We refer to a basic unit of deformation within the chain, the Dual-conformer, and consider the properties of a statistical system consisting of several Dual-conformers belonging to the same macromolecule but also to other macromolecules, all submitted to the constraints of a statistical model, the Grain-Field Statistics Theory, that defines their evolving interactive coupling as an open dissipative system [4,7,18]. The assumptions of the model define mathematically what “evolving interactive coupling” means when the full collection of Dual-conformers is brought out of equilibrium by an outside “deformation” (thermal or mechanical) to determine how many Dual-conformers of a certain type dynamically cooperate in an active system at any instant, how many systems are active and how many relax, and where the cooperative Dual-conformers are located: on a single chain or on several chains. The physics of dealing with all the chains at once in the statistics, redefining the coupling between the covalent and the inter-molecular interactions, is the model that we have adopted to describe the deformation of polymer melts and solids, above T_g_ and below T_g_ [7,8,9,18]. The theory not only addresses the spatial conformation of the Dual-conformers (cis, gauche, trans) but also their type (either b or F), defining at minimum six possible Dual-conformer “thermokinetics states”. The deformation of a set of macromolecules is quantified by the determination of the distribution of the total number of Dual-conformers among the thermokinetics states and by the determination of the strain and the stress generated by the evolution of the population in those states due to external constraints imposed on the collective system. The single dissipative system that assembles all the Dual-conformers from all the macromolecules is called “a single-dual-phase” system, the duality coming from the b or F character of the Dual-conformers (which is also the reason for the “Dual” connotation). In the Dual-Phase model of rheology the chains are there but are not the statistical systems (they can become the statistical system but only above T_LL_), and the phenomenon of entanglement, although initiated by an increase of the length of the chain, is not due to a variation in the sequence of various molecular events, say reptation vs. Rouse, happening to the chain deformation, but to the instability of the single dual-phase solution representing the statistical system of Dual-conformers interacting as a single system. At the critical molecular weight of the chain, M_c_, corresponding to the triggering of the instability of the single dual-phase, the system of single dual-phase conformers splits into two sub-systems of dual-phases, phase 1 and phase 2, to release from saturation the dissipative energy of the collective system of interactions. We call phase 1 and phase 2 the “Cross-Dual phases”, the word “cross” indicating that the two phases are themselves inter-coupled and dissipative. The split at M_c_ can be considered “a statistical fold” occurring at the scale of the collective level of the single dual-phase system; it could be imagined similar to the division of a biological cell into two biological cells, although in the case of the split of a single dual-phase the remaining “phases-cells” are not independent clones, they are interactively coupled. Note that at the local scale of the Dual-conformer dynamics, the definition of the b or F status for its Dual-conformers is equivalent to splitting the population of the Dual-conformers in type F and type b conformational states to form a single dual-phase dissipative system, In a sense, this split is “an internal fold” of the system, another form of duality, explaining the terminology of “Dual-Phase” for the dissipative systems just described, which are stable for M < M_c_ [4,7,8,9]. We have symbolized the “internal split” leading to a single Dual-Phase system of Dual-conformers by: [(b/F) ↔ (c,g,t)] and the “the external split” of a single Dual-Phase leading to two cross-dual-phases is symbolized in Equation (8):

(8) $$\begin{array}{l} {}[(b/F)↔(c,g,t)]\;\;↔\;\;[(b/F)↔(c,g,t){]}_{1}\cap [(b/F)↔(c,g,t){]}_{2}\\ \mathrm{SINGLE}\;\mathrm{DUAL-PHASE}\;\;\;\;\;\;\mathrm{CROSS-DUAL-PHASE} \end{array}$$

where b and F refer to an intermolecular bonding “conformation”, and (c,g,t) (i.e., cis, gauche, trans) refers to an “intra-chain conformation bonding” [7,8,9]. Figure 22 is a cartoon sketch of a single Dual-Phase system of Dual-conformers forming b-grains locally interfacing with F-conformers around them (left) and a Cross-Dual-Phase system where two different rheological states of b-grains surrounded by F-conformers coexist and are made stable (below T_LL_) by compensation of their enthalpic and entropic thermokinetic terms to generate an elastic dissipative shear wave (right) [7,18].

The dissipative statistics equations of a system of Dual-conformers can be simulated (the “EKTOR” simulator) to characterize the interactions between the Dual-conformers of a single chain, and it is shown that the chain can assume the shape of a macro-coil, see (a) in Figure 22, when the number of Dual-conformers in the macromolecule reaches a certain value, estimated around 42 for PS (our value for M’_c_ in Figure 2, Figure 3, Figure 4, Figure 5, Figure 6, Figure 7 and Figure 8). The interpenetration of 3 of these macro-coils can also be simulated to see if the resulting state after full or partial penetration differs from the state of a “grown macro-coil” (i.e., a similar macro-coil but with bigger b-grains, for instance).

The stability of a dissipative system of Dual-conformers is controlled by the Dissipative term in the EKTOR set of equations, which is defined by the value of I_d_ = Ln(N_b_/N_f_), where N_b_ and N_f_ are the population of the b and f Dual-conformers irrespective of their (c,t,g) spatial conformation, Chapter 3 of [9] and Appendix A. When the length of the macromolecules interpenetrating as macro-coils is below a certain value, which we have assumed corresponds to the manifestation of M_c_, the simulation shows [8,18] that the one Dual-Phase solution is stable (Figure 22 left box (b) state), i.e., its state does not differ from the rheological state of a grown system with a greater number of units in the total system population. However, the simulation shows that I_d_ grows exponentially towards an asymptote. At M_c_ and beyond, a two-single system is created (Figure 22 right box (c)) to reduce the value of I_d_ in the two dual phases created. This split of the original single Dual-Phase system into a more stable Cross-Dual-Phase system is what we suggest corresponds to the manifestation of the entanglements in polymer melt rheology.

In this Dual-Phase comprehension of entanglements, the thermodynamic aspect of the stability of the interactions is responsible for its occurrence. The value of M_c_ can be changed by manipulating externally-mechanically and thermally- the constraints on the Dual-Phase system, and this new conception of the entanglements gave birth to the technology of Rheo-Fluidification of melts to control their viscosity by “disentanglement” manipulation [4,7]. The Rheo-extrusion of hundreds of kilos of “sustained-oriented” pellets demonstrated that the “disentangled melts” produced by reheating the treated pellets exhibiting “Sustained-Orientation” behaved in a way incomprehensible to an interpretation of melt deformation based on the classical reptation relaxation time explanation.

We call the “thermokinetics parameters” of the Dual-Phase dissipative statistics the value of the population of the b and F states and the (cis, gauche, trans) states of the Dual-conformers statistics. These values can be obtained by simulation [8], assuming certain values for the statistical constants [9], or by Depolarization spectroscopy [9], but not directly by rheological experimental evidence. The simulation of the basic equations by the EKTOR simulator [8,9] permitted to define the strain produced by the system and quantify the corresponding stress generated by the system when submitted to strain rate deformations at various T for various M. This simulation work was capable of predicting all the rheological results found for a typical amorphous melt with M < M_c_: the proportionality of the viscosity with M (Equation (4), the Vogel-Fulcher dependence of the viscosity with T (Equation (3)), the effect of pressure, and the shear-thinning decrease of the viscosity at higher shear rates [7]. This success [8] proved that it was indeed possible to correlate the viscosity of the polymer melts to the population thermokinetic parameters. The remaining important issue is to find an empirical formulation of the viscosity measured experimentally so that we have access, directly or indirectly, to the thermokinetic parameters of the model. This research is ongoing and currently presents different possible solutions, one of which is presented here for the first time. In the criteria considered to achieve a satisfactory empirical formulation of the experimental viscosity dependence on T, M, the strain-rate and pressure, we have assumed that we could rely on our cartoon representations of the dissipative interactions in Figure 22 to define the b-grains “conformational enthalpy” and the “dynamic free volume” surrounding the b-grains [7,18]. In that regard, the formulation of the viscosity presented by Equations (6) and (7) offers the advantage of being able to extract from the fitting parameters pseudo thermodynamic quantities, ΔH_1_(M,T), ΔS_1_(M,T) for phase 1, and ΔH_2_(M,T), ΔS_2_(M,T) for phase 2, which qualitatively look like they could be related to the Dual-Phase thermokinetic parameters extracted from Figure 22. The enthalpies are clearly visible in Equation (7), and the entropies can be calculated by “entering” inside the exponent the values of the pre-exponential factors: b_2_ and b_4_ or b’_2_ and b’_4_. The other advantage of this empirical formula is that it can be applied to both the case of one single Dual-Phase (for M < M_c_) and to the case of two Dual-Phases (for M > M_c_), since the presence of the second phase, which is the mathematical clone of the first phase with different fitting parameters, can be eliminated by forcing the regressions to determine the first phase constants only. The veracity of our statement that a single exponential term fits better the M < M_c_ data range and that the M > M_c_ requires two exponential terms (to achieve the same level of fitting accuracy) can easily be proven by following the variation of the r^2^ and S_yx_ of the regressions in both the M < M_c_ range and the M > M_c_ range. Finally, to explain the choice of an exponential function, e.g., (a_1_exp(a_2_/T) + a_3_), instead of a hyperbolic function such as the Vogel-Fulcher Fulcher equation (Equation (3)), to describe the temperature dependence of Log μ_o_(T) at M given, we have already introduced before [10,11] the notion of “isomorphic functions”, which provide identical curves passing through the same data set when they are both used to fit those data. For usual rheological data, from, say, T_g_ to T_g_ + 150°, the Log μ_o_(T) vs. 1/T fitted by the exponential growth function provides the isomorph of Log μ_o_(T) vs. A + B/(T − T2) with an r^2^ difference smaller than 10^−5^ in most cases (i.e., with an uncertainty less than the experimental error to determine the value of log μ_o_). In this paper, our choice of the empirical function to describe the effect of T is the exponential growth function (or its Dual version above M_c_), but in other instances [11,12], our choice was to modify the Vogel-Fulcher equation to make its potential thermodynamic attributes ΔH and ΔS [11,12].

**Figure 23:**
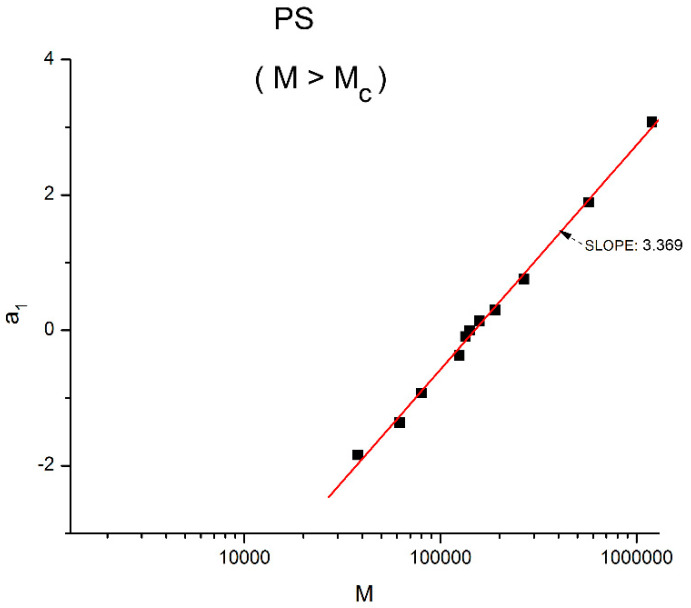
The M > M_c_ data are fitted with Equations (4) and (5), the classical formula. The best regressional fit of a_1_ vs. Log M has slope 3.369 and intercept −17.444 (r^2^ = 0.9982, S_yx_/DoF = 0.0883), which should be compared to the fit of the same data using M instead of logM and fitted according to the Cross-dual-phase model of the entanglement melt using a double exponential terms function.

The classical interpretations of the viscosity results are “molecular dynamic models”, based on the description of the properties of the macromolecular chain during flow and in particular of the molecular mechanisms occurring to the chain that produce the strain and require the stress needed for the chain motion. In other words, the classical models of rheology try to correlate the viscosity to the chain molecular description and to its evolution [1,5,6]. For instance, for the entangled melt of Figure 23, the attention is on understand how the molecular weight of the chain, actually M to the power 3.4, can be calculated and thus understood from molecular parameters arising from the chain’s description: from its length, from its location among its neighboring chains, from its displacement, its stretching, its relaxation time, etc. Let us critically examine if this power equation description of the viscosity could be understood from a Dual-Phase viewpoint.

**Figure 24:**
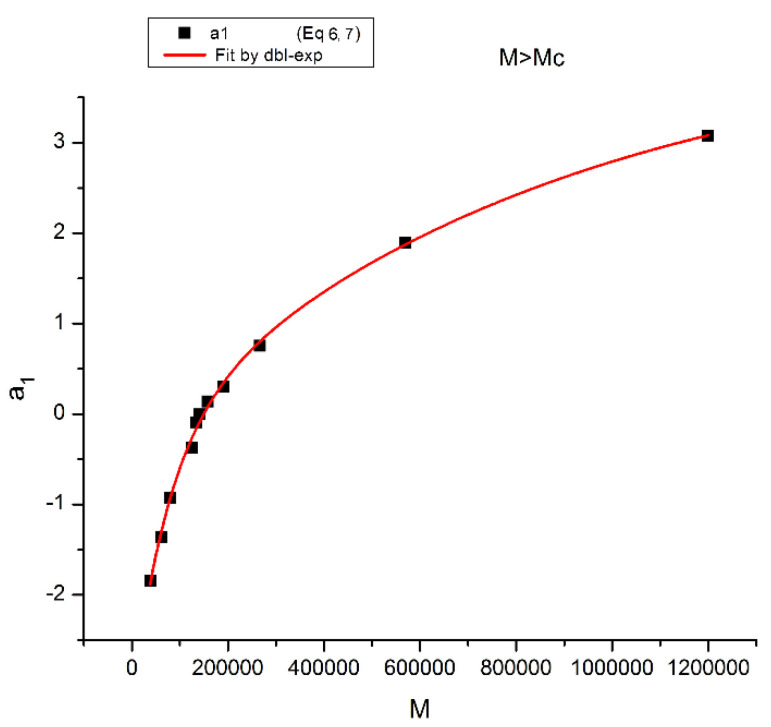
Same data as in Figure 23, replotted as a function of M and fitted by regression analysis using a double-exponential decay function of M: with the following two terms (red line): exp(-M/844,461) and exp(-M/75,380). r^2^ = 0.9981 S_yx_/DoF = 0.0064. This graph demystifies the use of the power exponent 3.4 to describe the dependence of viscosity on the molecular weight and contributes to the validation of the existence of two Cross-Dual-Phases for entangled melts [8].

The classical models are interested in determining in a plot like in Figure 1 whether the slope of log μ_o_ vs. log M is 1 or 3.4 to know if the polymer is un-entangled or entangled, and the Dual-Phase model needs to determine whether only a single or two Dual-Phases are present in the melt. Figure 23 definitely affirms that the melt is entangled in view of its power exponent equal to 3.37. The data in Figure 23 is re-plotted in Figure 24 to use a linear scale for M instead of a logarithmic scale, log M. The red curve passing through the points is obtained by fitting the linear scale data with a pair of exponential terms of M, i.e., exp (-M/M_1_) and exp(-M/M_2_), which could be attributed to the presence of two Dual-Phases and thus to an entangled melt (M > M_c_) pursuant to the Cross-Dual-Phase criterion. We find M_1_ = 75,380 ~ 2 M_c_ and M_2_ = 844,461. The regression by the pair of exponential terms of M in Figure 23 is at least as good, perhaps even slightly better, than the classical power law of Figure 23 (see the captions of Figure 23 and Figure 24). Now, if we can fit the M < M_c_viscosity data with a single dual-phase instead of 2, i.e., with a single exponential of M, this could be considered a simple validation of the single vs. cross dual-phase criterion for entanglements. Figure 2, Figure 3, Figure 4, Figure 5, Figure 6, Figure 7 and Figure 8 of section A show that the “un-entangled” state of the melt, below M_c_, is not as simple as a set of parallel lines with slope 1 when the temperature varies, which is what would be expected from the classical view. In fact, we saw that the location of the entanglement molecular weight, M_c_, could not be ascertained from the break of the viscosity extrapolated from the entanglement and the un-entanglement sides. However, if we select the highest temperature of the melt, T = 210 °C, corresponding to Figure 2, we see that the points below M_c_ make up a continuous curve without a break, which can be re-plotted (not shown) against M instead of logM, like it was done in Figure 23, and fitted by regression with either a single exponential of M or with two exponential terms of M: we verify that the fit is better with a single exponential term (r^2^ = 0.99873) than with 2 exponential terms (r^2^ = 0.9931), although the opposite is true for melts above M_c_, for which the 2 exponential terms solution is better. One could conclude from these simple validation tests, involving the difference between a regression using one exponential or two exponential terms of M, that the Dual-Phase explanation of entanglements is satisfied. The reality is unfortunately more complex, and a convincing proof requires a much more sophisticated analysis [8]. Nevertheless, we want to emphasize that the use, in Figure 23, of a different analytical formula to describe the molecular weight dependence of the viscosity of a polymer melt in lieu of the sacrosanct formula that uses the power exponent in Figure 23 represents a first step to demystify this misleading classical formula of melt rheology (Equations (4) and (5). In our opinion, these formulas are empirical expressions with a true value limited to engineering purposes only. The various attempts to attribute a physical sense to these mathematical expressions, especially for M > M_c_ (e.g., by the reptation school adepts [6]), are only theoretically justified if the predictions of the theory succeed in explaining all the other experimental facts, such as the fragmentation of the rheological ranges for M and T, as an example, or the “sustained-Orientation” of Rheo-fluidified melts, as a second example [10].

The formulation of the viscous flow behavior of M > M_c_ melts from the Cross-Dual-Phase perspective offers new arguments regarding the reptation model historical dilemma: while the original de Gennes’s theory predicted a power exponent of 3 for the molecular weight dependence of Newtonian melt viscosity [6], several authors successfully tweaked the mathematics of the initial reptation model to explain the “reality”, i.e., that viscosity appeared to follow a 3.4 exponent behavior instead of 3. This is discussed in [4,7,10]. Are the dual-phase results of Figure 15 and Figure 16 actually suggesting that the original reptation exponent of 3, calculated by de Gennes, adequately describes the viscosity in phase 2, the core phase of the Cross-Dual-phase model, and that the coupling with the “entangled phase”, phase 1, is responsible for the correction to the power exponent, not the tube fluctuations nor any other possible tweaking due to molecular reasons?. In the (0,0) configuration Cross-Dual-Phase treatment of viscosity, we find that viscosity is the product of two terms, each M and T dependent. At high T, one of the terms varies with M with a power exponent 3, while the other term varies with M with a power exponent 0.373, for PS. The slope 3 for phase 2 is what the Dual-Phase model predicts at a high T, just like what de Gennes projected starting from molecular dynamics considerations. We show in Refs. [4,9,11,12] that for T > T_LL_, which is the case in Figure 15 and Figure 16, the Dual-Phase dissipative term, I_d_, relaxes out, making the statistics converge with the non-dissipative Boltzmann’s statistics, rendering legit both the computations of de Gennes and the cross-dual phase explanations of the results. This is the reason why, under such conditions of equivalence, i.e., for T > T_LL_ only, we expect to find for phase 2 the de Gennes’s slope of 3 when the log μ_o2_, calculated from b_1_, b_2_, b_4_ in Equation (7) is plotted like in Figure 16. If the equivalence between de Gennes’s calculations and the Dual-Phase approach is justified at higher T, one may question the utility of the subsequent improvements by many authors to correct the original calculations of de Gennes: were they actually necessary?

As far as the Dual-Phase model of the entanglement is concerned, the requirement to have two Cross-dual-phases for M > M_c_, and simply one dual-phase for M < M_c_ is a theoretical statement from a theory, hypothetically predicting what changes at M_c_. But the parameters entering the theory are not directly available, and we cannot validate it directly. But the consequence of having a split of the system at M_c_ guides us in the choice of the empirical formula that may describe the experimental facts: the single exponential growth function of 1/T has a r^2^ close to 1 when fitting all the data for M < M_c_, yet starts to deteriorate in fitting quality around M_e_ and becomes questionable, even poor, at M_c_; the addition of another exponential growth term for the M > M_c_ data makes r^2^ raise again to almost perfection. These empirical formulas are “inspired” by the theoretical model of a split at M_c_ and describe the facts well, although they do not directly address the issues of the stability of a single phase or a cross-dual-phase dissipative system, which are the true theoretical issues that explain the cause of the split at M_c_. The “Dissipative function” of the EKTOR simulations cannot be measured directly from rheological data, but its assumed existence and the ability to simulate its relaxation behavior continue to inspire new perspectives about the origin of M_c_ with, as a consequence, the so-called Rheo-Fluidification (aka“disentanglement”) and Sustained-Orientation technologies [4,7,8]. Theoretically, in our view, the possibility to create disentangled states in polymer melts to reduce favorably their processing viscosity is due to the inducement, by the effect of shear and vibration, of the instability of the entanglement state of the melt. This instability results from the consequence of the effect of the Grain-Field Statistics on the interplay between the size of the system of interactions (related to M), its incidence on the b-grain population (vs. the dynamic free volume), and the value of the thermokinetic constants of the dissipative open system [7,8,9]. An explication of the Rheo-Fluidification of melts to produce Sustained-Orientation does not seem possible using the current understanding of entanglements by the current paradigm of rheology [4,7].

## 4. Conclusions

There are sophisticated theories that have been advanced for the last 70 years that derive from the assumptions expressed by Figure 1 (Rouse [5] and reptation [6], for instance). Actually, all the theories from the past regarding viscous flow have assumed the separation of the effect of M and T in the expression of viscosity. It is classically admitted [1] that the (Newtonian) viscosity depends on the product of two parameters: a friction factor which is controlled solely by local features such as the free volume, itself a function of T only, and a molecular weight dependent factor which is a function of the configuration of the chains, itself controlled by M. The difference between the various theories arose from the different statistical treatments of the molecular weight factor of the macromolecules. This was the case for the molecular dynamic theories of Rouse [5] applicable to melts with M < M_c_ and of de Gennes [6] for the reptation model applicable to entangled melts with M > M_c_. The failure of both the Rouse and the reptation models to adequately describe the experimental data has been suggested in another publication [4]. In this paper, we summarize the results of a thorough mathematical analysis of viscosity data on a series of monodispersed PS samples [2,3,7,8,10] to test the separation of M and T in the expression of the viscosity equations.

The different approach in this paper is the use of a mathematical formula in which M and T do not separate to express the dependence of M and T on the melt viscosity; it succeeds to fit the data points “perfectly”, at least as well as the classical formula. However, it concludes the opposite of what the classical formula claims regarding the separation of M and T, which is summarized in Figure 1. Our paper analyzes the relative impact of the fitting parameters magnitude on the residual curves of the regressions when M or T varies and concludes that the features in Figure 1 are only valid for certain values of M and T, otherwise are only approximations. The classical formulation of viscosity, Equations (3)–(5) of the Development section, may be good “isomorphs” of our new formulation, Equations (6) and (7), in the data range chosen, leading to wrong conclusions when trying to understand them theoretically, or it could be the other way around, our contradictors could rightfully say. For instance, our new empirical equation of viscosity generates data that can be plotted like in Figure 1, validating the classical slopes of 1 and 3.4 when those data are fitted pursuant to the classical formula. However, the understanding of the new viscosity equation by the Dual-Phase and Cross-Dual-Phase concepts is possible and induces a very different understanding of entanglements at M_c_. This ambiguity of having two distinct mathematical formulas provide similarly satisfactory descriptions of the experimental results, making the two formulas “isomorphs”, can be resolved by considering the consequences of the two different theoretical approaches and the possibility to test experimentally their projections. In that sense, the new rheological formula proposed projects the fragmentation of the rheological ranges defined by the effect of M and the effect of T, thus predicting the existence of “new” transitions in the melt, T_g_ + 23 and T_LL_ for temperature, M_c_, 4M_c_ and M’_c_ = M_c_/8 for the molecular weight. The classical approach does not predict the existence of such fragmentation of the rheological ranges, nor does it understand its origin. The other projection of the new viscosity equation is the “period-doubling” of the values of the molecular weights that lead to the true separation of the effect of T and M:

Our contradictors may ask why so many experienced scientists could have missed the possibility that T and M did not separate in the viscosity expression. The use of the log-log plot in Figure 1 was possibly one of the reasons for the visual success impression that may have misled the scientific community in the belief that the separation of M and T was validated [10]. Another possible reason was the coherence with previous work and the simplifications the separation of T and M implied theoretically: the influence of the molecular weight of the chains could be treated like in the rubber theory, by considerations on the statistical configuration of the chains [1], and, separately, the influence of temperature was explained by the free volume, successfully addressing the non-Arrhenius behavior [1,10]. In reality, the main reason why these results were not apparent to previous investigators is explained in this paper: for T above approximately 448 °K (175 °C), the Newtonian viscosity of the PS melts demonstrates an apparent satisfactory classical behavior for all M > 2M_c_, which is the usual range tested. The mathematical testing of the separation of M and T explains that the apparent validation becomes systematically worse as T and M are decreased. By re-plotting the viscous data according to the classical paradigm of rheology (Figure 1) at various temperatures (Figure 2, Figure 3, Figure 4, Figure 5, Figure 6, Figure 7 and Figure 8), we reveal that the current definition of M_c_ by the break of the viscosity curves across M_c_, does not validate that M_c_ is independent of T, again defaulting the assumption of the separation of M and T. The problem is that, in general, rheologists test and report high-temperature data only and rarely (if not ever) show the evolution of their Figure 1 at several lower temperatures, as we did (Figure 2, Figure 3, Figure 4, Figure 5, Figure 6, Figure 7 and Figure 8). In summary, the separation of T and M in the expression of viscosity was an illusion that was hard to reveal. The realization that it was an illusion and is actually not true has consequences on the understanding of viscoelasticity: for instance, it implies that the time-temperature superposition principle (TTS) is itself invalid, a conclusion also validated by the fragmentation of the rheological ranges for M and T. A second consequence of the illusion of the separation of M and T is that it precisely hides the existence of the fragmentation of the molecular range with its period doubling structure (Figure 19), one of the most interesting differences between the classical and the dual-phase views of the melt rheology. Overall, the current understanding of entanglements is challenged by the results presented in this communication. However, as is concluded below, a word of caution must be added.

In previous communications [4,7], we pointed to convincing Rheo-SANS experimental evidence challenging the predictions of the popular reptation model of polymer melt deformation and concluded that the mechanism of reptation of the macromolecules through a set of topological obstacles was probably inadequate to describe the concept of entanglements and of shear-thinning. In this paper, we show that a new way to analyze classical data raises intriguing new questions regarding the effect of molecular weight and temperature on viscosity, taking us far, very far, from a molecular dynamic explanation of the rheology of melts. We conclude once more [4] on the inadequacy of the current modelization of the physical properties of polymers [5,6,19] and on the need to change the paradigm.

We briefly presented in the paper the basics of the “Dual-conformers” Dissipative Statistics, which assumes that Dual-conformers are the statistical units of the macromolecules that need to be characterized, not the single macromolecules themselves, to understand the properties of a set of macromolecules responding collectively to a deformation (thermal or mechanical). This new approach to the interactions in polymers, which is described in more detail in previous publications [7,9,18], is summarized in Figure 22 to explain the possible physical reason behind the appearance of the M’_c_ transition in Figure 2, Figure 3, Figure 4, Figure 5, Figure 6, Figure 7 and Figure 8, as the formation of stable macro-coils, and to visualize the split of a single Dual-Phase system into a Cross-Dual Phase system at M_c_, providing for M_c_, the entanglement molecular weight, a very different explanation from the current admitted view. It will be shown in future presentations [8] that the new Dual-Phase and Cross-Dual-Phase model not only proposes a new understanding of “entanglements” but also of the dynamic melt properties G′(ω,T) and G″(ω,T) and the normal stresses. It predicts shear-thinning and strain softening in shear mode and strain-hardening in extensional mode; it successfully describes the transitional behavior at T_g_, from a solid-like to a liquid-like behavior, predicts [11,12] the existence and the characteristics of the T_LL_ upper melt transition temperature as the end of dissipative modulation in a Dissipative system [9]. Finally, the theory addresses the stability (or the strain induced lack of stability) of the Cross-Dual Phase entanglement network, leading to the Rheo-Fluidification technologies [7].

The theoretical assumptions of the new model and the quantitative descriptions it generates constitute a whole new understanding of the viscoelastic properties of polymers that could be considered the premises of a new paradigm in that field of physics. However, our last word must address a word of caution necessary before we can generalize these conclusions to the status of a new reality. Our model is in its infancy and in constant improvement. What we found in this paper is, indeed, quite unexpected and intriguing: the effect of T and M combine to create a fragmentation of the rheological ranges resulting in a periodical validation of the criteria for variable separation, but only at discrete specific values of M that verify period doubling at M_c_/2: M_c_, 2M_c_, 4M_c_, etc.). We believe that such an important conclusion (Figure 19) is based on experimental results that should be duplicated and even extended using more modern automated instruments, permitting the use of a greater number of samples. We suggest tripling the current set of 22 samples to cover the same range of molecular weight (from M = 550 to M = 1.2 Million g/mole). We also suggest repeating the rheological data using a dynamic rheometer instead of a viscometer operating in the linear visco-elastic range and covering the temperature span from (T_g_ + 10) to (T_g_ + 110), spacing the temperature range every 5 °C to obtain the maximum number of points on the temperature scale.

In conclusion, the results of a new analysis of the Newtonian data for a series of monodispersed PS samples raise more fundamental questions regarding the validity of the current established views in polymer physics.

## Data Availability

Data are contained within the article.

## Appendix A

(Notions of Grain-Field-Statistics)

Dual-Conformers

3 to 5 successive covalent bonds of the macromolecular chain define a Dual-conformer. Their spatial conformational state can be cis, gauche, trans, which can be reduced to 2: cis-gauche (“cg”) and trans (“t”) to simplify. Dual-conformers also have a dissipative conformational state. Their dissipative state can be “f” or “b” (free or bonded inter-molecularly).

The duality of the Dual-conformers implies that we split each state cg and t into an F and b category, giving 4 states cgf, cgb, tf, tb; the result is a dual partition, between cg and t units on one hand, and between the F and b units on the other hand. The two partition functions are coupled by kinetic equations and energy equations. The energy equation includes a Dissipative term I_d_ = Ln (N_b_/N_f_) where N_b_ is the total number of b dual-conformers and N_f_ is the total number of f dual-conformers. I_d_ collapses to zero at equilibrium.

The Dual-Split Dissipative equations (the EKTOR model) present the most basic assumptions controlling the dynamics of a dissipative system of Dual-conformers in interactions. The new equations converge to traditional kinetic equations at long times or under “true” equilibrium conditions. Under non-isothermal conditions, triggered by heating or cooling, the system of interactions becomes self-dissipative, and the conformational duality is responsible for a structure of the Free Energy.

Mechanical “straining” of the dynamic systems of interactive dual-conformers, i.e., favoring an increase of the population of the ‘t” conformers in the population statistics, requires to apply a stress to the system. The stress can be quantified from the modification of (Δ_e_ − Δ_eo_) by the non-equilibrium states, where Δ_eo_ is the conformational energy difference between a flexed (cg) and a trans (t) spatial conformation at equilibrium.

Activated and Relaxed Open Dissipated Systems.

The dynamics of a single dissipative system of Dual-conformers is essential but not sufficient to describe the plurality of mechanical modes of deformation available to a dissipative strained system. In the Grain Field Statistics approach of mechanical deformation, one is concerned with all the possible organizational changes occurring to the Dual-conformers statistics by application of a stress field. This includes the possibility to generate a network of open dissipative systems where some systems are actively stretched (S) while other are Relaxing (R). The deformation of a polymeric material consists of a change in the partition function of the conformers via a dynamic structuration of the interactions into Stretched (Δ_e_ ≠ Δ_eo_) and Relaxing (Δ_e_ = Δ_eo_) open dissipative systems.

The process of activation (S)—deactivation (R) can be viewed as a fluctuating grain field in the temporal and space dimensions.

Open Dissipative Network Dynamics of Dual-Conformers.

-A Dual-Split Dissipative Equations (Δ_e_ = Δ_eo_): (B_or_ type)

N_b_ = n_tb_ + n_cgb_
N_f_ = n_tf_ + n_cgf_
B_o_ = (N_b_ + N_f_) (A1)

dn_tb_/dt = 0.5dN_b_/dt − k_1_*n_tb_ + k_2_*(N_b_ − n_tb_)
dn_tf_/dt = 0.5dN_f_/dt − k_1_*n_tf_ + k_2_*(N_f_ − n_tf_)
k1 = ν_m_ exp(−Δ_1_/T)
k2 = ν_m_ exp(−Δ_2_/T) Δ_m_ = (Δ_1_ + Δ_2_)/2 Δ_eo_ = (Δ_1_ − Δ_2_)/2 (A2)

Ln (n_tb_/n_cgb_) + Ln (n_tf_/n_cgf_) + Ln (N_b_/N_f_) = 2 Ln (k_2_/k_1_) = 4Δ_eo_/T (A3)

(Note: the gas constant is equated to 1)

I_d_ = Ln(N_b_/N_f_) is the dissipative term.

Note: Equations (A1)–(A3) apply to “B_or_” systems (Δ_e_ = Δ_eo_) and correspond to an internal split of B_o_ into N_b_, N_f_) to generate the inter mode of dissipation.

In addition to the Dual-Split Kinetics constants (Δ_m_, ν_m_ and Δ_e_), the study of the mechanical properties of a polymer requires the knowledge of:

(1) The amount of strain induced by the rotation of a conformer from one spatial conformation to the other.
(2) The relationship for a stretched system between the kinetic stretch rate, Rs, and (Δ_e_ − Δ_eo_).
(3) The mechanical behavior of open “stretched” and “relaxed” systems, i.e., during and after it is submitted to a given deformation.

-B Mechanically Disturbed “Stretched” Systems (Bos type).

l = “kinetic length”

l = n_tb_ + n_tF_ (total population of t-conformers)

Additional constraint: R = [dntb/dt + dntf/dt] is imposed

Stress is determined from (Δ_e_ − Δ_eo_) but is not proportional to it.

Three cases:

- B_os_ is constant (closed system)
- B_os_ can vary (open system)
- B_os_ varies such that the Global dissipative Free energy of Bo remains minimum: (open dissipative system)

The B_os_ systems structure with respect to the Bor systems (external split):

B_o_ = B_os_(t) + B_or_(t).

-C Compensation Dynamics.

The interplay between B_or_(t) and Δ_e_(t) where B_o_ = B_or_(t) + B_os_(t), the dynamic split, is determined at each time by the minimization of the global dissipative function. The variation of (Δ_e_(t) − Δ_eo_) is correlated to the stress produced by the deformed statistical system.
