# Correction: Ibar, J.P. Raising Two More Fundamental Questions Regarding the Classical Views on the Rheology of Polymer Melts. *Polymers* 2024, *16*, 2042

**DOI:** 10.3390/polym16213029

**Published:** 2024-10-29

**Authors:** Jean Pierre Ibar

**Affiliations:** Rheology Department, Polymat Institute, University of the Basque Country (UPV/EHU), 20018 Donostia (St Sebastian), Gipuskoa, Spain; jpibar@alum.mit.edu

There was an error in the original publication [1]. It contains a false Equation (8). There is the middle component [(b/f) ↔ (c,g,t)] on the right end side should be deleted. There should only be two bracket terms [(b/F) ↔ (c,g,t)]_1_ ∩ [(b/F) ↔ (c,g,t)]_2_ on the right end side.

A correction has been made to the equation in the Section 4.

(8) $$\begin{array}{l} {}[(b/F)↔(c,g,t)]\;\;↔\;\;[(b/F)↔(c,g,t){]}_{1}\cap [(b/F)↔(c,g,t){]}_{2}\\ \mathrm{SINGLE}\;\mathrm{DUAL-PHASE}\;\;\;\;\;\;\mathrm{CROSS-DUAL-PHASE} \end{array}$$

where b and F refer to an intermolecular bonding “conformation”, and (c,g,t) (i.e., cis, gauche, trans) refers to an “intra-chain conformation bonding” [7–9].

The authors state that the scientific conclusions are unaffected. This correction was approved by the Academic Editor. The original publication has also been updated.
